# Recovery cycle times of inferior colliculus neurons in the awake bat measured with spike counts and latencies

**DOI:** 10.3389/fncir.2012.00056

**Published:** 2012-08-20

**Authors:** Riziq Sayegh, Brandon Aubie, Siavosh Fazel-Pour, Paul A. Faure

**Affiliations:** Department of Psychology, Neuroscience & Behaviour, McMaster UniversityHamilton, ON, Canada

**Keywords:** auditory neurophysiology, big brown bat (*Eptesicus fuscus*), duration-tuned neurons, echolocation, neuroethology, paired tone stimulation

## Abstract

Neural responses in the mammalian auditory midbrain (inferior colliculus; IC) arise from complex interactions of synaptic excitation, inhibition, and intrinsic properties of the cell. Temporally selective duration-tuned neurons (DTNs) in the IC are hypothesized to arise through the convergence of excitatory and inhibitory synaptic inputs offset in time. Synaptic inhibition can be inferred from extracellular recordings by presenting pairs of pulses (paired tone stimulation) and comparing the evoked responses of the cell to each pulse. We obtained single unit recordings from the IC of the awake big brown bat (*Eptesicus fuscus*) and used paired tone stimulation to measure the recovery cycle times of DTNs and non-temporally selective auditory neurons. By systematically varying the interpulse interval (IPI) of the paired tone stimulus, we determined the minimum IPI required for a neuron's spike count or its spike latency (first- or last-spike latency) in response to the second tone to recover to within ≥50% of the cell's baseline count or to within 1 SD of it's baseline latency in response to the first tone. Recovery times of shortpass DTNs were significantly shorter than those of bandpass DTNs, and recovery times of bandpass DTNs were longer than allpass neurons not selective for stimulus duration. Recovery times measured with spike counts were positively correlated with those measured with spike latencies. Recovery times were also correlated with first-spike latency (FSL). These findings, combined with previous studies on duration tuning in the IC, suggest that persistent inhibition is a defining characteristic of DTNs. Herein, we discuss measuring recovery times of neurons with spike counts and latencies. We also highlight how persistent inhibition could determine neural recovery times and serve as a potential mechanism underlying the precedence effect in humans. Finally, we explore implications of recovery times for DTNs in the context of bat hearing and echolocation.

## 1. Introduction

The ability to extract temporal information from an acoustic signal is important for processing human speech (Denes, 1955), discriminating species-specific communication calls (Pollack and Hoy, 1979), localizing sounds (Knudsen and Konishi, 1979; Carr and Konishi, 1990), and echolocation by bats (Simmons, 1971, 1979; Suga and O'Neill, 1979; O'Neill and Suga, 1982). Neurons with spiking responses selective for signal duration, known as duration-tuned neurons (DTNs), provide one neural mechanism for encoding temporal information. DTNs have been observed across a variety of vertebrate species and taxa including frogs (Potter, 1965; Gooler and Feng, 1992; Leary et al., 2008), chinchillas (Chen, 1998), guinea pigs (Wang et al., 2006), mice (Brand et al., 2000; Xia et al., 2000; Tan and Borst, 2007), rats (Pérez-González et al., 2006), cats (He et al., 1997), and bats (Jen and Schlegel, 1982; Pinheiro et al., 1991; Casseday et al., 1994; Ehrlich et al., 1997; Fuzessery and Hall, 1999; Faure et al., 2003; Mora and Kössl, 2004; Luo et al., 2008). Because DTNs have also been found in the visual cortex of cats (Duysens et al., 1996), this suggests that neural mechanisms of duration selectivity may be similar across sensory modalities. The physiological response properties and underlying neural mechanisms of auditory DTNs have been studied most extensively in echolocating bats. The biological function(s) of DTNs to hearing is(are) unknown; however, the range of neural best durations (BD) within a species (BD = stimulus duration that causes maximum spiking) correlates with the range of vocalization durations of species-specific communication sounds in non-echolocating vertebrates and with biosonar pulse durations in echolocating bats (for reviews, see Sayegh et al., 2011; Jen et al., 2012).

Duration tuning in the mammalian inferior colliculus (IC) is hypothesized to arise from the convergence and temporal interaction of excitatory and inhibitory synaptic inputs arriving at the neuron (Casseday et al., 1994, 2000; Covey et al., 1996; Faure et al., 2003; Aubie et al., 2009). Biologically plausible computational models of DTNs support the hypothesis that neural mechanisms of duration selectivity may be shared across vertebrates (Aubie et al., 2012). A number of studies have demonstrated that neural inhibition is necessary for creating DTNs in the IC. For example, focal application of GABA_A_ and/or glycine receptor antagonists have been shown to greatly diminish and/or eliminate the spiking responses of DTNs (Fuzessery and Hall, 1999; Jen and Feng, 1999), with the blocking of GABA_A_ receptors having the greatest effect on temporal tuning and duration selectivity (Casseday et al., 2000). Whole-cell intracellular patch clamp recordings (Covey et al., 1996; Tan and Borst, 2007; Leary et al., 2008) and/or single-unit extracellular recordings combined with paired tone stimulation (Faure et al., 2003) have revealed that inhibitory inputs to DTNs usually precede excitatory inputs, and that the inhibition lasts from 5 to 150 ms after stimulus offset. Hence, DTNs receive inhibition that leads excitation. This inhibition also persists for as long or longer than the duration of the stimulus that evoked it. We know that inhibition occurs in some types of IC neurons that are not tuned to stimulus duration (Faingold et al., 1991; Pollak and Park, 1993; Torterolo et al., 1995; Kuwada et al., 1997; Pedemonte et al., 1997; Klug et al., 1999), and in these cells inhibition can persist for as long as 100 ms after stimulus offset (Yin, 1994; Covey et al., 1996; Litovsky and Delgutte, 2002). Owing to the importance of inhibition in creating the temporal tuning profile and response properties of DTNs, we hypothesized that the leading and persistent inhibition evoked by each signal in a paired tone stimulus could temporally interact and sum, resulting in DTNs exhibiting longer recovery cycle times than non-DTNs. Herein we test this hypothesis.

The recovery time of an evoked neural response can be measured with paired pulse stimulation (Grinnell, 1963). Often the stimulus is a pair of equal amplitude pure tones set to the cell's characteristic or best excitatory frequency (BEF) and presented at varying interpulse intervals (IPIs). Some studies have used pairs of acoustic clicks presented at varying interstimulus delays (Fitzpatrick et al., 1995; Litovsky and Yin, 1998a; Litovsky and Delgutte, 2002), while others have used pairs of frequency modulated (FM) sweeps or variable amplitude tones to mimic the loud outgoing vocalizations and faint returning echoes used for echolocation by bats (Grinnell, 1963; Suga, 1964; Friend et al., 1966; Pollak et al., 1977a,b; Lu et al., 1997; Wang et al., 2010). A cell's recovery time is measured by determining the minimum IPI required for the spiking response evoked by the second stimulus (R2) to recover within a specified level of the spiking response evoked by the first stimulus (R1). The most common and unbiased criterion for measuring recovery time is to report the IPI where the R2/R1 ratio is ≥0.5. Note that this measure is normally based enitrely on spike counts. To the best of our knowledge, no previous study has measured neural recovery times with a spike latency criterion.

Previous studies in bats reported that spike count recovery cycle times of IC neurons are highly variable, ranging anywhere from 4 to 200 ms (Suga, 1964; Friend et al., 1966; Suga and Schlegel, 1973; Pollak et al., 1977a; Lu et al., 1997; Tang et al., 2011). The recovery cycle times of many DTNs are shortest when the pulse and echo durations are set to the cell's BD and presented at biologically relevant pulse-echo amplitude differences (Wang et al., 2008, 2010). The frequency selectivity of DTNs has also been reported to sharpen when the pulse and echo are presented at BD (Wu and Jen, 2008b). These findings suggest that the responses of DTNs may be specialized for processing loud outgoing echolocation pulses and fainter returning echos. Because DTNs are found in both echolocating and non-echolocating mammals, the ability to echolocate cannot be a prerequisite for the evolution of auditory duration selectivity (Sayegh et al., 2011). Nevertheless, this does not preclude a more specialized functional role for DTNs in hearing and echolocation by bats.

Recovery cycle times also provide a way to observe the effects of synaptic inhibition to a neuron. Application of bicucculine, a GABA_A_ receptor antagonist, has been shown to shorten the recovery times of IC neurons (Lu et al., 1997; Zhou and Jen, 2003), suggesting that inhibitory inputs control the minimum time needed for response recovery. In this study, we measured and compared the recovery cycle times of DTNs and non-DTNs using both spike count and spike latency measures as a way to further our understanding about the strength and time course of the leading and persistent inhibition that is responsible for the creation of auditory midbrain microcircuits sensitive to temporal acoustic features.

## 2. Methods

### 2.1. Surgical procedures

Electrophysiological data were obtained from 33 adult big brown bats (*Eptesicus fuscus*) of both sexes that were housed in a husbandry facility where colony temperature and lighting varied according to ambient conditions (Faure et al., 2009). To facilitate multiple recordings and to precisely replicate the position of the bat's head between sessions, a stainless steel post was glued to the skull. Prior to the head-posting surgery, bats were given a subcutaneous injection of buprenorphine (0.03 mL; 1:9 mixture of 0.3 mg/mL Temgesic and sterile water; 0.045 mg/kg). For the surgery, bats were first placed in an anesthesia induction chamber (12 × 10 × 10 cm) where they inhaled a 1–5% isofluorane:oxygen gaseous mixture (flow rate: 1 L/min). Anesthetized bats were then placed in a foam-lined body restraint within a stereotaxic alignment system (David Kopf Instruments Model 1900) fitted with a custom mask for gas inhalation. The hair covering the skull was shaved and the underlying skin was swabbed with Betadine® disinfectant. Local anesthetic (0.2 mL bupivicaine; 5 mg/mL) was injected subcutaneously prior to making a midline incision in the scalp. The temporal muscles were reflected, the skull was scraped clean and swabbed with 70% ethanol, and a post was glued to the skull overlying the cortex with cyanoacrylate adhesive (Henkel Loctite Corporation) cured with liquid acrylic hardener (Zipkicker; Pacer Technology®). A chlorided silver wire, attached to the head post, was placed under the temporal muscles and served as the reference electrode. Recordings began 1–4 days after surgery. Each bat was used in 1–8 sessions lasting ca. 4–8 h each on separate days. Recordings were terminated if a bat showed signs of discomfort (e.g., struggling body movements). Between sessions, the electrode penetration site was covered with a piece of contact lens and Gelfoam® coated in Polysporin®. Bats were housed individually in a temperature- and humidity-controlled room and were given *ad libitum* access to food and water. All procedures were approved by the McMaster University Animal Research Ethics Board and were in accordance with the Canadian Council on Animal Care.

### 2.2. Electrophysiological recordings

Recordings were conducted inside a double-walled, sound attenuating booth with electrical shielding (Industrial Acoustics Co., Inc.). Prior to recording, each bat was given a subcutaneous injection of a neuroleptic (0.3 mL; 1:1 mixture of 0.05 mg/mL fentanyl citrate and 2.5 mg/mL Inapsine [droperidol]; 19.1 mg/kg). Bats were then placed in a foam-lined body restraint that was suspended by springs within a small animal stereotaxic frame that was customized for bats (ASI Instruments) and mounted atop of an air vibration table (TMC Micro-G). The bat's head was immobilized by securing the headpost to a stainless steel rod attached to a manipulator (ASI Instruments) mounted on the stereotaxic frame. The dorsal surface of the IC was exposed for recording by making a small hole in the skull and dura mater with a scalpel. Single-unit extracellular recordings were made with thin-wall borosilicate glass microelectrodes with a capillary filament (o.d. = 1.2 mm; A-M Systems, Inc.) and filled with 3 M NaCl. Typical electrode resistances ranged from 10 to 30 MΩ. Electrodes were positioned over the dorsal surface of the IC with manual manipulators (ASI Instruments), and advanced into the brain with a stepping hydraulic micropositioner (David Kopf Instruments Model 2650). Action potentials were recorded with a Neuroprobe amplifier (A-M Systems Model 1600) whose 10× output was bandpass filtered and further amplified (500–1000×) by a Tucker Davis Technologies spike pre-conditioner (TDT PC1; lowpass *f*_*c*_ = 7 kHz; high-pass *f*_*c*_ = 300 Hz). Spike times were logged on a computer by passing the PC1 output to a spike discriminator (TDT SD1) and then an event timer (TDT ET1) synchronized to a timing generator (TDT TG6). Electrodes were visually aimed at the dorsal surface of the IC and all recordings are assumed to be from the central nucleus of the IC (ICc).

### 2.3. Stimulus generation and data collection

Stimulus generation and on-line data collection were controlled with custom software that displayed spike times as dot raster displays ordered by the acoustic parameter that was randomized during unit testing (see Faure et al., 2003). Briefly, pure tone pulses were digitally generated with a two-channel array processor (TDT Apos II; 357 kHz sampling rate) optically interfaced to two digital-to-analog (D/A) converters (TDT DA3-2) whose individual outputs were fed to a low-pass anti-aliasing filter (TDT FT6-2; *f*_*c*_ = 120 kHz), two programmable attenuators (TDT PA5) and two signal mixers (TDT SM5) with equal weighting. The output of each mixer was fed to a manual attenuator (Leader LAT-45) before final amplification (Krohn-Hite Model 7500). All stimuli were presented monaurally, contralateral to the IC being recorded, using a Brüel & Kjær (B&K) $\frac{1}{4}$ inch condenser microphone (Type 4939; protective grid on) modified for use as a loudspeaker with a transmitting adaptor (B&K Type UA-9020) to correct for non-linearities in the transfer function (Frederiksen, 1977). The loudspeaker was positioned ca. 1 mm in front of the external auditory meatus. The output of each speaker, measured with a B&K Type 4138 $\frac{1}{8}$ inch condenser microphone (90° incidence; grid off) connected to a measuring amplifier (B&K Type 2606) and bandpass filter (K-H Model 3500), was quantified with a sound calibrator (B&K Type 4231) and expressed in decibels sound pressure level (dB SPL re 20 μPa) equivalent to the peak amplitude of continuous tones of the same frequency (Stapells et al., 1982). The loudspeaker transfer functions were flat ±6 dB from 28–118 kHz, and there was at least 30 dB attenuation at the ear opposite the source (Ehrlich et al., 1997). All stimuli had rise/fall times of 0.4 or 0.5 ms shaped with a square cosine function and were presented at a trial stimulation rate of 3 Hz.

Single units were found by searching with short duration pure tones and/or downward FM sweeps. Upon unit isolation, we determined the cell's BEF, acoustic threshold, first-spike latency (FSL) and last-spike latency (LSL) re signal onset, and for DTNs we also determined the BD and duration-selective response class (i.e., shortpass, bandpass or longpass DTNs; see Faure et al., 2003; Fremouw et al., 2005). In all cases, neural response parameters were determined by systematically varying the frequency, attenuation, or duration of the stimulus in blocks, with 10–20 stimulus repetitions per randomized step in each block. Following the paired tone stimulation paradigm of Faure et al. (2003), we presented cells with pairs of BEF tone pulses. The onset of the first pulse (P1) was fixed in time relative to the onset of recording; the onset of the second pulse (P2) was systematically varied. The stimulus IPI was defined as the time between the onset of P1 and the onset of P2. For DTNs the durations of P1 and P2 were set to the cell's BD, whereas for non-DTNs the paired tone duration was randomly chosen from 1–9 ms. Because P1 and P2 were matched in stimulus frequency, duration, amplitude, and phase at all IPIs presented, whenever the two tones temporally overlapped they summed to form a single composite tone with a +6 dB amplitude pedestal, the duration of which was determined by the amount of overlap.

### 2.4. Measuring recovery times

We tested 73 IC neurons with paired tone stimulation and generated 132 data files: 59 cells were tested at both +10 dB and +20 dB above threshold (118 files), and 14 cells were tested at only +10 dB above threshold. The IPI between P1 and P2 was randomly varied, typically in 2 ms steps (115 of 132 files; 87%); however, two files used 1 ms steps, 1 file used 2.5 ms steps, 1 file used 4 ms steps, and 13 files used 5 ms steps.

#### 2.4.1. Baseline data and windowing responses

**Figure 1:**
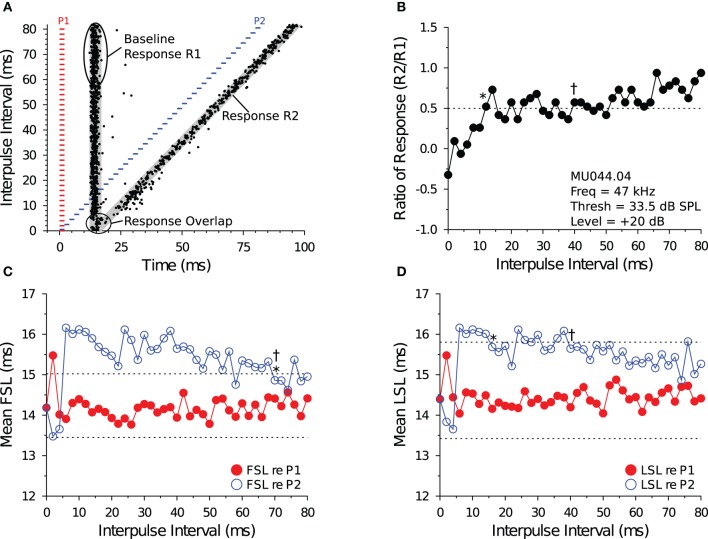
**Measuring recovery cycle times of IC neurons. (A)** Dot raster display of an *in vivo* shortpass DTN in response to paired tone stimulation at varying IPIs. The *red bars* represent the onset, duration, and offset of the first tone pulse (P1), and the *blue bars* represent the onset, duration, and offset of the second tone pulse (P2). Stimuli P1 and P2 were set to the cell's BEF and, if duration-tuned, BD. The timing of action potentials are illustrated as *black dots*. We calculated the mean ± SD baseline FSL and baseline LSL in response to P1 over the 10 longest IPIs from the onset of P1 to the onset of P2. Baseline latencies were used to define the P1 and P2 analysis windows (*gray regions*) used for assigning spikes as being evoked by stimulus P1 or P2, and for calculating the Baseline Response R1. The P1 analysis window began at the onset of P1 + baseline FSL − 2 SDs and ended at the onset of P1 + mean LSL + 2 SDs (re P1). The P2 analysis window began at the onset of P2 + baseline FSL − 2 SDs and ended at the onset of P2 + mean LSL + 2 SDs (re P2), and was used to measure the responses evoked by P2 (Response R2). For trials where the P1 and P2 analysis windows overlapped, a single analysis window was defined starting from the onset of P1 + baseline FSL − 2 SDs and ending at the onset of P2 + baseline LSL + 2 SDs. **(B)** Recovery times measured with spike count. The ordinate shows the spike count ratio of response (R2/Baseline R1) as a function of stimulus IPI. The *dotted line* at 0.5 represents the 50% spike count recovery threshold. The asterisk (*) at 12 ms illustrates the recovery time of the cell determined with the short-to-long method, and the dagger (†) at 40 ms represents the recovery time of the same cell determined with the long-to-short method. **(C,D)** Recovery times measured with spike latency. The *red line with closed symbols* represents spike latency (re P1), and the *blue line with open symbols* represents spike latency (re P2). The *dotted lines* represent ± 1 SD relative to the mean baseline FSL or baseline LSL (see **A**). **(C)** Mean FSL (re P1 and P2) as a function of IPI. The asterisk (*) at 70 ms illustrates the FSL recovery time measured with the short-to-long method, and the dagger (†) at 70 ms represents the FSL recovery time of the same cell determined with the long-to-short method. **(D)** Mean LSL (re P1 and P2) as a function of IPI. The asterisk (*) at 16 ms illustrates the LSL recovery time of the cell measured with the short-to-long method, and the dagger (†) at 40 ms represents the LSL recovery time of the same cell determined with the long-to-short method. In this example, FSL took longer to recover than LSL. MU044.04.09: BEF, 47 kHz; BD, 2 ms; threshold, 33.5 dB SPL; amplitude +20 dB re threshold; 20 repetitions per IPI step.

For each file, we measured the baseline FSL and baseline LSL in response to tone P1 for the 10 trials with the longest IPIs. We did this to minimize the influence of tone P2 on the measurement of the Baseline Response R1 re P1 (see Figure 1A). We used 20 stimulus repetitions at each IPI to calculate a mean ± standard deviation (SD) FSL and LSL (re P1). We then averaged the 10 means and 10 SDs to calculate a grand mean ± average SD baseline FSL and baseline LSL (re P1) for each file. The baseline FSL and baseline LSL were used to define the P1 and P2 analysis windows. The P1 analysis window started at the onset of P1 + baseline FSL −2 SDs and ended at the onset of P1 + baseline LSL +2 SDs. The P2 analysis window started at the onset of P2 + baseline FSL −2 SDs and ended at the onset of P2 + baseline LSL +2 SDs. Spikes were assumed to be evoked by P1 if they fell into the P1 analysis window, and spikes were assumed to be evoked by P2 if they fell into the P2 analysis window. For trials where the IPI was small and the P1 and P2 analysis windows overlapped—when the onset of P1 + baseline LSL +2 SDs was > the onset of P2 + baseline FSL −2 SDs, thus making it difficult to confidently assign spikes as being evoked by either P1 or P2—we used a single, combined analysis window to measure the evoked response. The combined analysis window started from the onset of P1 + baseline FSL −2 SDs and ended at the onset of P2 + baseline LSL +2 SDs.

#### 2.4.2. Spike count recovery

We used the P1 and P2 analysis windows (described above) to count spikes and measure spike latencies evoked by tones P1 and P2, respectively, and to calculate the response criteria for measuring recovery cycle times. We did this so that evoked responses were compared with equal duration analysis windows. Assuming spike latencies are normally distributed, then at 2 SDs wide the P1 and P2 analysis windows should capture ≥95% of evoked responses. The P1 analysis window was used to measure the Baseline spiking Response R1 evoked by tone P1. The P2 analysis window was used to measure the spiking Response R2 evoked by tone P2 (Figure 1A). At each IPI, we calculated a R2/Baseline R1 ratio of response (Figure 1B). The R2 spike count was defined as having “recovered” when this ratio was ≥0.5—that is, when the spike count evoked by P2 recovered to within 50% of the baseline spike count evoked by P1. For trials where the IPI was small and the P1 and P2 analysis windows overlapped, the spike count ratio of response was calculated by first counting the number of spikes that fell into the combined P1 + P2 analysis window and then subtracting the Baseline Response R1 spike count before dividing this value by the Baseline Response R1 spike count, which is similar to the method used by Suga (1964) to deal with response overlap.

Some cells showed variation (ringing) in their spike count ratio of response function, so we developed two algorithms to measure recovery times from the functions. The algorithms assess response recovery starting from different ends of the function (Figure 1B). The *short-to-long method* assesses the function starting from the shortest IPIs on the left and moving toward the longest IPIs on the right. Using this technique, the recovery time of a cell was defined as the smallest IPI where the ratio of response function crossed and remained ≥0.5 for at least two consecutive IPIs (see ^*^ in Figure 1B). In a small subset of files (9/132, 6.8%), we observed a brief facilitation in the ratio of response function at short IPIs, followed by a decrease below 0.5 at intermediate IPIs, and then an increase above 0.5 at longer IPIs (e.g., Figures 6C,D). For these files, the recovery time was defined as the smallest IPI where the spike count ratio of response function remained ≥0.5 for most of the remaining points at longer IPIs (based on visual inspection by 2 observers), which is similar to the method used by Fitzpatrick et al. (1995). The *long-to-short method* assesses the spike count recovery function starting from the longest IPIs on the right and moving toward the shortest IPIs on the left. With this technique the recovery time of a cell was defined as the largest IPI where the spike count ratio of response function was ≥0.5 if at least two consecutive data points at smaller IPIs were <0.5 (see † in Figure 1B), or if most data points at shorter IPIs remained below 0.5 (based on visual inspection by 2 observers).

#### 2.4.3. Spike latency recovery

In most data files we noticed an increase in the FSL or LSL (or both) of the evoked response (re P2) at short IPIs, and then a return to baseline R1 latencies at longer IPIs (e.g., Figures 1C,D). We used this latency change as an alternative method for determining the recovery times of IC neurons. Using the Baseline Response R1 FSL and LSL data (re P1) for each file, we measured response recovery with spike latencies using a ±1 SD criterion. We then employed similar short-to-long and long-to-short analysis algorithms to measure spike latency recovery times. The *short-to-long method* starts with the shortest IPI on the left where the R2 latency had increased to >1 SD above the Baseline Response R1 latency, and moving right it selects the shortest subsequent IPI where the R2 latency returns and remains within 1 SD of baseline for at least two consecutive IPIs (see ^*^ in Figures 1C,D). The *long-to-short method* starts from the longest IPI on the right and moving left it determines the shortest subsequent IPI where the R2 latency falls within 1 SD of baseline if at least two consecutive points at shorter IPIs were >1 SD of baseline (see † in Figures 1C,D). A number of data files did not show a ±1 SD change in FSL (8/132, 6.1%) or LSL (56/132, 42.4%), and for these cases it was not possible to measure a spike latency recovery time.

### 2.5. Data analysis

Unless stated otherwise, all data are reported as the mean ± standard error (SE). Parameter correlations were calculated with linear regression in Python (SciPy module) and relationships are reported as the coefficient of determination (*R*^2^ and *p*-values). Unless explicitly testing for factor effects, we grouped recovery time values across cell types (bandpass, shortpass, allpass) at both +10 dB and +20 dB (re threshold). We used the linear and non-linear mixed-effects models analysis-of-variance (ANOVA) package written in R to test for effects of cell type, analysis method, and relative amplitude on recovery cycle times, FSLs, and LSLs, with cell type and relative amplitude as fixed factors and cell ID as a random factor (Pinheiro and Bates, 2000).

## 3. Results

### 3.1. Response properties

DTNs can be categorized into one of three response classes based on the shape of the duration tuning function (Sayegh et al., 2011). Bandpass DTNs respond maximally at BD, with spike counts that eventually fall to ≤50% of the maximum at durations both longer and shorter than BD. Shortpass DTNs also respond maximally at BD, with spike counts that eventually fall to ≤50% of the maximum at durations longer than BD but not shorter. The spiking responses of shortpass and bandpass DTNs are typically transient and offset-evoked, with FSL (re stimulus onset) increasing with stimulus duration (Faure et al., 2003). Longpass DTNs do not have a BD; instead, they respond only when the stimulus duration exceeds some minimum duration (Faure et al., 2003; Aubie et al., 2009; Sayegh et al., 2011). Longpass DTNs differ from typical sensory neurons in that they do not show a decrease in FSL with increasing stimulus amplitude that is typical of neurons that integrate stimulus energy (Brand et al., 2000; Faure et al., 2003; Pérez-González et al., 2006). Longpass DTNs were not used in this report. By definition, allpass neurons are not duration-selective and therefore do not have a BD. Allpass neurons spike in response to all signal durations that contain sufficient stimulus energy. The response pattern of allpass neurons can be transient or sustained, with spikes typically occurring at a constant (onset-evoked) FSL re stimulus onset.

We obtained single unit extracellular recordings from 73 IC neurons. Of these, 16 (22%) were shortpass DTNs, 18 (25%) were bandpass DTNs, and 39 (53%) were not duration-selective (i.e., allpass neurons). Recovery times were determined at +10 dB for all cells and at +20 dB re threshold for 59 cells (14 shortpass, 13 bandpass, and 32 allpass).

**Figure 2:**
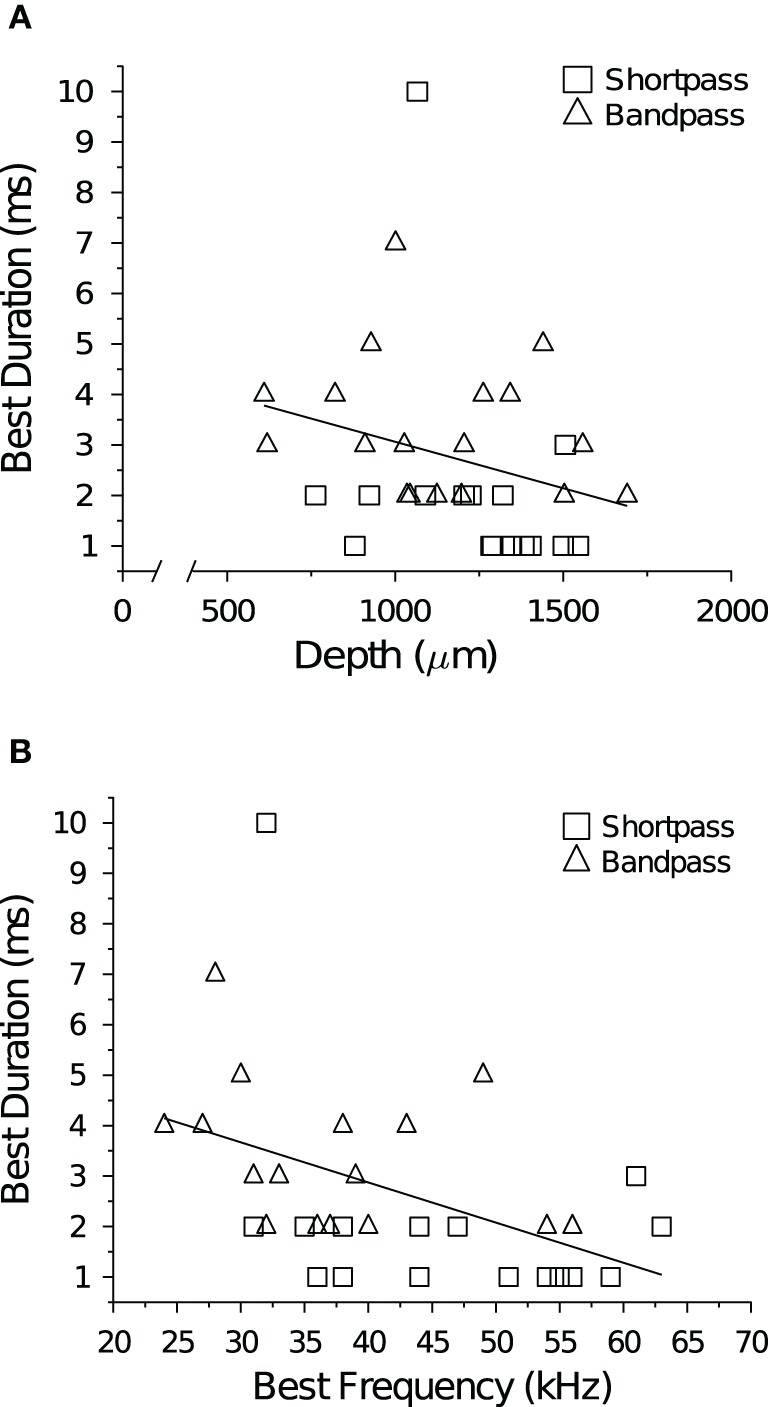
**Temporal tuning and spatial topography or tonotopy in the IC. (A)** There was no correlation between neuronal BD and the depth of the recording electrode (*R*^2^ = 0.070, *p* = 0.13). Note the broken abscissa. **(B)** There was a negative correlation between neuronal BD and BEF (*R*^2^ = 0.20, *p* = 0.0081).

Traveling dorsal to ventral in direction, the BEF of cells systematically increased as the depth of the recording electrode was advanced into the IC (*R*^2^=0.57, *p* « 0.001; data not shown). This tonotopic relationship held true within all cell types and response classes (allpass *R*^2^ = 0.65, *p* « 0.001; shortpass *R*^2^ = 0.56, *p* « 0.001; and bandpass *R*^2^ = 0.41, *p* < 0.001). There was no correlation between neuronal BD and electrode depth (Figure 2A), but there was a significant negative correlation between BD and BEF (Figure 2B). There was also no correlation between BEF and the mean baseline spike count at +10 dB (*R*^2^ = 0.030, *p* = 0.15), but at +20 dB (re threshold) there was a weak, positive correlation (*R*^2^ = 0.068, *p* = 0.046).

**Figure 3:**
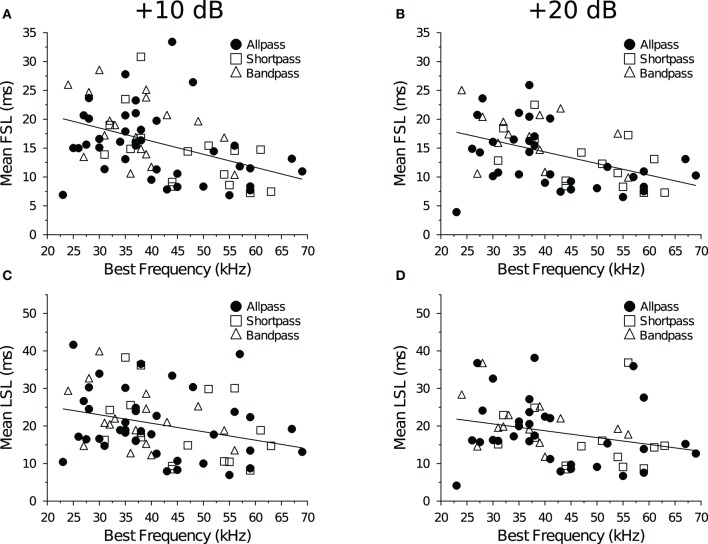
**Spike latency and tonotopy in the IC. (A,B)** First-spike latency as a function of BEF. Neurons with lower BEFs had longer average FSLs at both **(A)** +10 dB (*R*^2^ = 0.18, *p* = 0.00019) and **(B)** +20 dB (*R*^2^ = 0.21, *p* = 0.00031) re threshold. **(C,D)** Last-spike Latency as a function of BEF. Neurons with lower BEFs had longer average LSLs at both **(C)** +10 dB (*R*^2^ = 0.087, *p* = 0.011) and **(D)** +20 dB (*R*^2^ = 0.070, *p* = 0.044) re threshold.

We measured the average spike latency (re stimulus onset) at each level above threshold (Table 1) and found that FSL (*F* = 2.12, *p* = 0.13) and LSL (*F* = 0.17, *p* = 0.84) did not vary as a function of response class. Mean FSLs decreased with a +10 dB increase in stimulus amplitude (*F* = 7.46, *p* = 0.0084), but there was no change in LSL (*F* = 0.0016, *p* = 0.97). Neurons with higher BEFs had shorter FSLs (Figures 3A,B). There was also a negative correlation between BEF and LSL (Figures 3C,D). Neurons with longer FSLs (but not LSLs) were distributed more dorsally in the IC at shallower electrode depths (data not shown).

**Table 1:** **Mean ± SE first- and last-spike latency as a function of cell type and level above threshold**.

**Cell type**	***n***	**Spike latency (ms) +10 dB**	***n***	**Spike latency (ms) +20 dB**
**FIRST-SPIKE LATENCY**				
Allpass	39	15.42 ± 0.98	32	13.20 ± 0.96
Bandpass	18	18.33 ± 1.30	13	16.86 ± 1.28
Shortpass	16	14.32 ± 1.57	14	12.76 ± 1.24
**LAST-SPIKE LATENCY**				
Allpass	39	20.37 ± 1.43	32	18.42 ± 1.58
Bandpass	18	21.34 ± 1.75	13	20.71 ± 1.79
Shortpass	16	19.57 ± 2.49	14	16.00 ± 2.08

The average baseline R1 latency at +10 and +20 dB (re threshold) was used as the input for each cell.

### 3.2. Recovery cycle times

**Figure 4:**
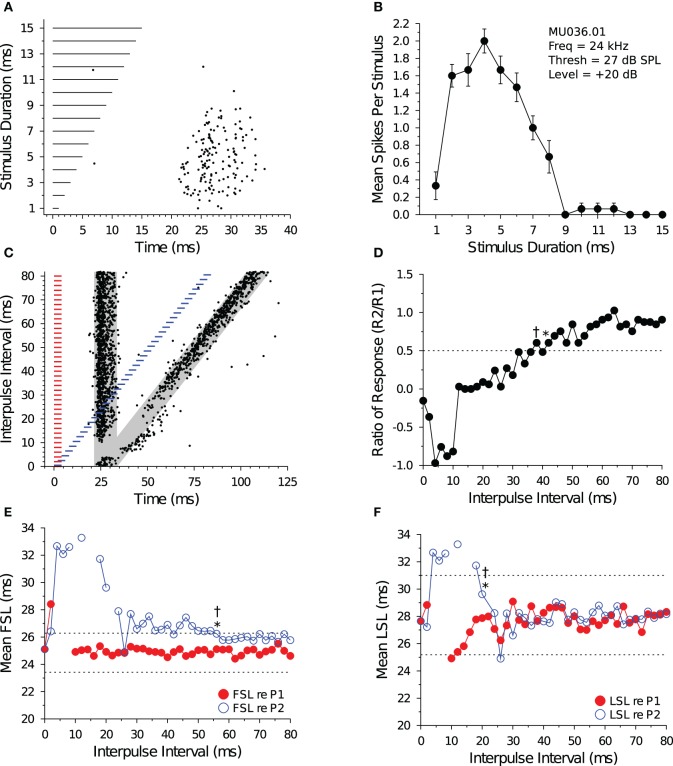
**Response and recovery in a bandpass DTN. (A)** Dot raster display of spiking in a bandpass DTN in response to variable duration BEF tones. **(B)** Mean ± SE spikes per stimulus as a function of stimulus duration. This cell had a BD of 4 ms. **(C)** Dot raster display illustrating spiking in response to pairs of BD tones presented at variable IPIs. The *shaded gray regions* illustrate the customized P1 and P2 analysis windows bounded by ±2 SDs from the baseline FSL and baseline LSL of the cell (see Figure 1). **(D)** Spike count ratio of response as a function of IPI. Evoked spiking in response to P2 recovers to within 50% of baseline (*dotted line*) in response to P1 at 42 ms using the short-to-long method (*), and at 38 ms using the long-to-short method (†). **(E)** Mean FSL and **(F)** mean LSL as a function of IPI for responses evoked by P1 (red closed symbols) and P2 (blue open symbols). **(E)** The cell's FSL (re P2) returns to within 1 SD of baseline at 56 ms using both the short-to-long and long-to-short methods, whereas **(F)** the LSL (re P2) returns to within 1 SD of baseline at 20 ms using both the short-to-long and long-to-short methods. Latencies determined after windowing responses with the cell-specific P1 and P2 analysis windows. *Dotted lines* represent ±1 SD relative to baseline latency (re P1). Missing values represent IPIs where no spikes fell into the analysis window. **(A,B)** MU036.01.06: BEF, 24 kHz; threshold, 27 dB SPL; amplitude +20 dB re threshold; 15 repetitions per stimulus. **(C–F)** MU036.01.31: BEF, 24 kHz; threshold, 27 dB SPL; amplitude +20 dB re threshold; 20 repetitions per IPI step.

Figure 4 illustrates the response characteristics of a bandpass DTN to presentations of variable duration BEF tones (Figures 4A,B) and to pairs of BEF and BD tones varied in IPI (Figures 4C,D). This cell responded to 24 kHz tone durations between 1 and 8 ms, with a maximum of ca. 2 spikes per stimulus at a BD of 4 ms. Because the cell's FSL increased with stimulus duration it was characterized as offset responding. When stimulated with pairs of BD tones that were randomly varied in IPI, spike counts in response to tone P2 became suppressed (Figure 4D) and the spike latency (both FSL and LSL) was delayed at short IPIs (Figures 4E,F). Evidence that neural inhibition alters the responses can be seen in Figure 4C during and following the period of response overlap. At an IPI of 2 ms, the leading inhibition evoked by each stimulus sums and this appears to lengthen the cell's FSL without drastically altering its spike count. At IPIs from 4 to ca. 36 ms, the persistent inhibition evoked by P1 appears to interact with the leading inhibition evoked by P2 and this suppresses the spike count and delays the FSL of the response evoked by P2. Eventually, the spike count and latencies recover to within baseline values, although subtle effects of persistent inhibition can still be observed at long IPIs because the mean FSL (re P2) does not begin to overlap the mean FSL (re P1) until an IPI of 76 ms. Indeed, for this cell it is clear that FSL takes longer to recover than LSL. The recovery time of the cell, as determined from the spike count ratio of response function, was similar for the short-to-long (42 ms) and long-to-short (38 ms) analysis methods. The recovery time of the cell, as determined from the FSL function (re P2), returned to within 1 SD of baseline at 56 ms using both the short-to-long and long-to-short methods (Figure 4E). The recovery time of the cell, as determined from LSL function (re P2), returned to within 1 SD of baseline at 20 ms using both methods (Figure 4F).

**Figure 5:**
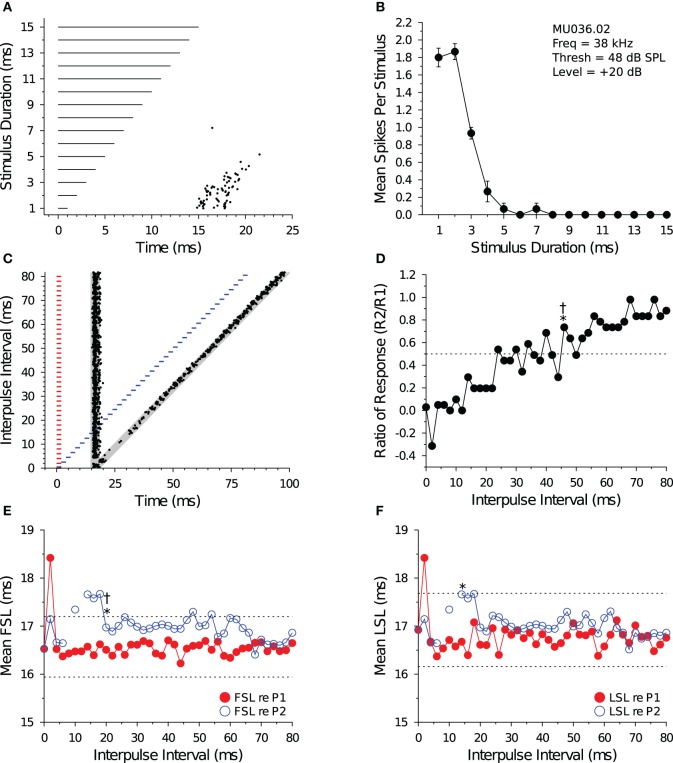
**Response and recovery in a shortpass DTN. (A)** Dot raster display of spiking in a shortpass DTN in response to variable duration BEF tones. **(B)** Mean ± SE spikes per stimulus as a function of stimulus duration. This cell had a BD of 2 ms. **(C)** Dot raster display illustrating spiking in response to pairs of BD tones presented at variable IPIs. The *shaded gray regions* illustrate the customized P1 and P2 analysis windows bounded by ±2 SDs from the baseline FSL and baseline LSL of the cell (see Figure 1). **(D)** Spike count ratio of response as a function of IPI. Spiking in response to tone P2 recovers to within 50% of baseline (*dotted line*) in response to P1 at 46 ms using both the short-to-long (*) and long-to-short (†) method. **(E)** Mean FSL and **(F)** mean LSL as a function of IPI for responses evoked by tone P1 (red closed symbols) and tone P2 (blue open symbols). **(E)** The cell's FSL (re P2) returns to within 1 SD of baseline at 20 ms using both the short-to-long and long-to-short methods, whereas **(F)** the LSL (re P2) returns to within 1 SD of baseline at 14 ms using the short-to-long method. No recovery time value was obtained with the long-to-short method because there was not two consecutive IPIs where the function deviated by >1 SD from the baseline LSL. Latencies determined after windowing spikes with the cell-specific P1 and P2 analysis windows. *Dotted lines* represent ±1 SD relative to baseline latency (re P1). Missing values represent IPIs where no spikes fell into the analysis window. **(A,B)** MU036.02.06: BEF, 38 kHz; threshold, 48 dB SPL; amplitude +20 dB re threshold; 15 repetitions per stimulus. **(C–F)** MU036.02.12: BEF, 38 kHz; threshold, 48 dB SPL; amplitude +20 dB re threshold; 20 repetitions per IPI step.

Figure 5 illustrates the response characteristics of a shortpass DTN to presentations of variable duration BEF tones (Figures 5A,B) and to pairs of BEF and BD tones varied in IPI (Figures 5C,D). This cell responded to 38 kHz tone durations between 1 and 4 ms, with a maximum of ca. 1.9 spikes per stimulus at a BD of 2 ms. The FSL of the cell also increased with stimulus duration and followed stimulus offset. When stimulated with pairs of BD tones that were randomly varied in IPI, the spike count in response to tone P2 became suppressed (Figure 5D) and both the FSL and LSL were delayed at short IPIs (Figures 5E,F). Eventually, the spike count and latencies recovered to within baseline values. The effect of neural inhibition can be seen in Figure 5C during and following the period of response overlap. At an IPI of 2 ms, when the paired BD stimulus was effectively a single 4 ms tone with a brief amplitude modulation at its midpoint (caused by the fall-and-rise times of each stimulus), the leading inhibition evoked by each stimulus sums and this appears to decrease the spike count and lengthen the FSL (re P2). At IPIs from 4 to ca. 40 ms, the persistent inhibition evoked by P1 appears to interact with the leading inhibition evoked by P2 and this suppresses the spike count and delays the FSL of the response evoked by P2. The recovery time of the cell, as determined from the spike count ratio of response function, was 46 ms for both the short-to-long and long-to-short analysis methods. The recovery time of the cell, as determined from the FSL function (re P2), returned to within 1 SD of baseline at an IPI of 20 ms for both analysis methods (Figure 5E). The recovery time of the cell, as determined from the LSL function (re P2), returned to within 1 SD of baseline at an IPI of 14 ms with the short-to-long method, and we were unable to measure a recovery time with the long-to-short method because LSL did not deviate by >1 SD for two consecutive IPIs (Figure 5F). Notice again that subtle effects of persistent inhibition can be observed in the paired tone responses because the mean FSL and mean LSL (re P2) do not begin to overlap the mean FSL and mean LSL (re P1) until IPIs longer than the measured recovery times. Again, FSL takes longer to recover than LSL.

**Figure 6:**
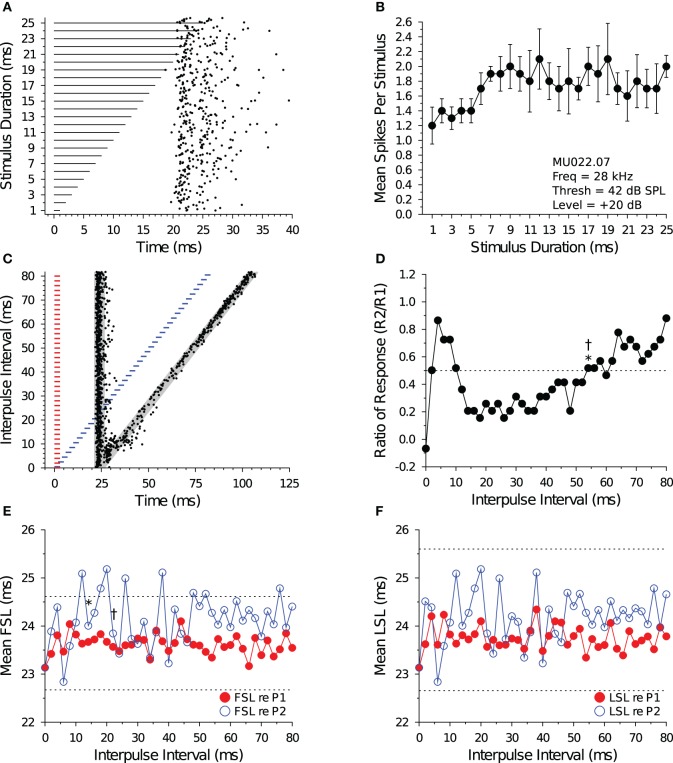
**Response and recovery in a non-DTN. (A)** Dot raster display of spiking in an allpass neuron in response to variable duration BEF tones. **(B)** Mean ± SE spikes per stimulus as a function of stimulus duration. By definition, allpass neurons do not have a BD. **(C)** Dot raster display illustrating spiking in response to pairs of 3 ms tones presented at variable IPIs. The *shaded gray regions* illustrate the customized P1 and P2 analysis windows bounded by ±2 SDs from the baseline FSL and baseline LSL of the cell (see Figure 1). **(D)** Spike count ratio of response as a function of IPI. Spiking in response to tone P2 recovers to within 50% of baseline (*dotted line*) in response to P1 at 54 ms using both the short-to-long (*) and long-to-short (†) method. **(E)** Mean FSL and **(F)** mean LSL as a function of IPI for responses evoked by tone P1 (red closed symbols) and tone P2 (blue open symbols). **(E)** The cell's FSL (re P2) returns to within 1 SD of baseline at 14 ms using the short-to-long method and at 22 ms using the long-to-short method, whereas **(F)** the LSL (re P2) function did not deviate by >1 SD from baseline at any IPI, hence no recovery time value was obtained. Latencies determined after windowing spikes with the cell-specific P1 and P2 analysis windows. *Dotted lines* represent ±1 SD relative to baseline latency (re P1). **(A,B)** MU036.02.06: BEF, 38 kHz; threshold, 48 dB SPL; amplitude +20 dB re threshold; 15 repetitions per stimulus. **(C–F)** MU036.02.12: BEF, 38 kHz; threshold, 48 dB SPL; amplitude +20 dB re threshold; 20 repetitions per IPI step.

Figure 6 illustrates the response characteristics of an allpass neuron to presentations of 28 kHz BEF tones that were randomly varied in duration (Figures 6A,B) and to pairs of BEF tones varied in IPI (Figures 6C,D). For this cell, spike counts remained within 50% of the maximum response at all durations tested. Because FSL did not change with stimulus duration, the cell was characterized as onset responding. When the cell was stimulated with 3 ms tone pairs that were randomly varied in IPI (Figure 6C), a brief facilitatory response was observed at IPIs from 2 to 10 ms before the spike count (re P2) became suppressed at intermediate IPIs (Figure 6D). Paired tone stimulation only slightly delayed FSLs re P2 (Figure 6E), and LSLs re P2 were unaffected (Figure 6F). The recovery time of the cell, as determined from the spike count ratio of response function, was 54 ms using both the short-to-long and long-to-short analysis methods. The recovery time of the cell, as determined from the FSL function (re P2), returned to within 1 SD of baseline at an IPI of 14 ms using the short-to-long method, and at 22 ms using the long-to-short method (Figure 6E). We were unable to measure a recovery time with LSL because it did not deviate by >1 SD at any IPI tested (Figure 6F).

### 3.3. Recovery cycles measured with spike counts and latencies

**Figure 7:**
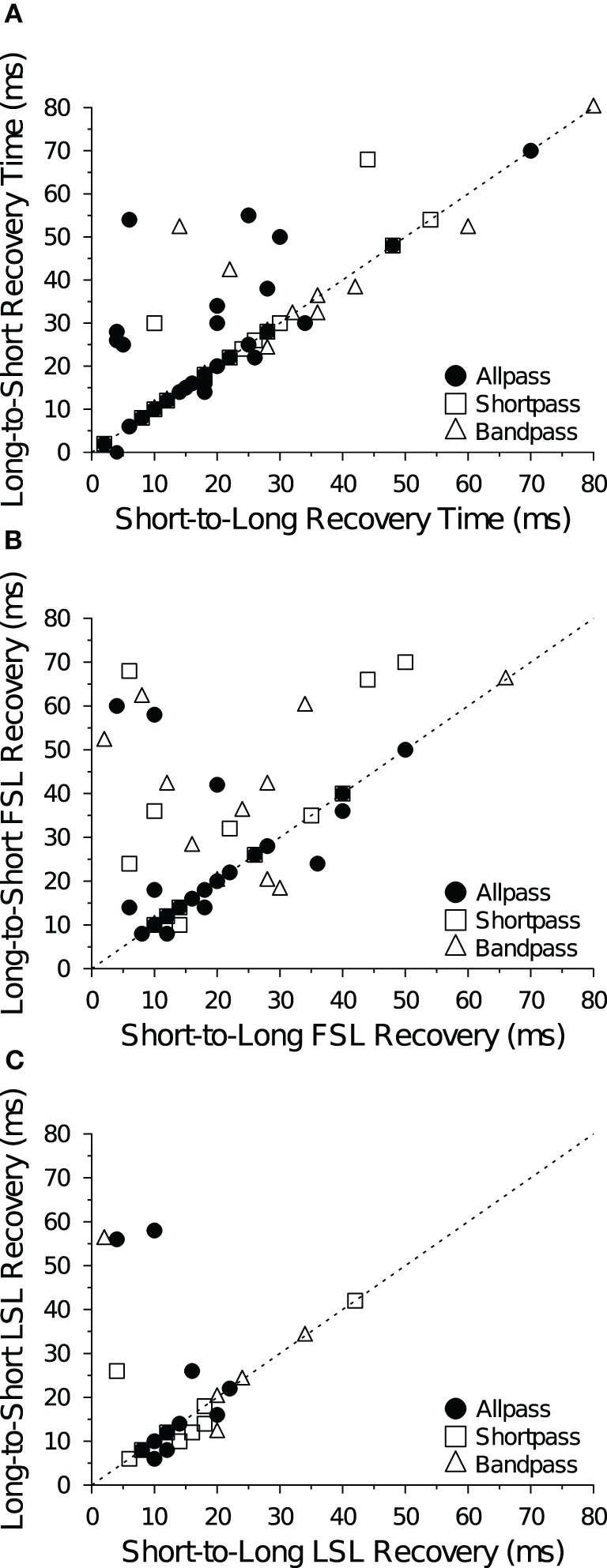
**Comparison of recovery time measures.** Each panel shows recovery times of IC neurons at +10 dB (re threshold) measured with the short-to-long (abscissa) and long-to-short (ordinate) analysis methods based on **(A)** spike count, **(B)** FSL and **(C)** LSL criteria. The two methods are in agreement when points fall along the *dotted unity line y* = *x*. Recovery times were positively correlated at +10 dB (A: *R*^2^ = 0.77, *p* « 0.001; B: *R*^2^ = 0.30, *p* « 0.001; C: *R*^2^ = 0.22, *p* = 0.0089) and at +20 dB (data not shown) above threshold. The sample size (*n*) and number of points that fall above, below (but not on) the unity line are: **(A)** *n* = 73, 14 above, 9 below; **(B)** *n* = 57, 19 above, 7 below; and **(C)** *n* = 30, 6 above, 7 below.

For the majority of cells and data files (92 of 132 files; 70%), recovery cycle times measured with the short-to-long and long-to-short analysis algorithms yielded identical values. Indeed, recovery times determined with the two algorithms were highly correlated at both +10 dB (Figure 7) and +20 dB (data not shown) above threshold for spike counts (Figure 7A), FSL (Figure 7B) and LSL measures (Figure 7C). Of the remaining 40 files that were not in agreement, 15 had recovery times that differed by ≤4 ms (absolute value). When the two methods for determining recovery times did not agree, the long-to-short method generally yielded longer values than the short-to-long method, as evidenced by the number of points falling above the unity lines in Figure 7.

Table 2 lists the mean ± SE 50% spike count recovery times determined with both analysis algorithms as a function of neural response class and level above threshold. Using the short-to-long method, non-duration-selective neurons and shortpass DTNs had the shortest recovery times, and bandpass DTNs had significantly longer recovery times (short-to-long spike recovery time: *F* = 4.31, *p* = 0.017). When we analyzed the data measured with the long-to-short technique, the differences in recovery cycle times across neural response classes were no longer significant (long-to-short spike recovery time: *F* = 1.68, *p* = 0.1942); however, a statistically significant difference in recovery cycle times between neural response classes re-emerged when the data were re-analyzed using the average recovery cycle time of the short-to-long and long-to-short algorithms (average spike recovery time: *F* = 3.23, *p* = 0.046). There was no effect of tone amplitude (level above threshold) on recovery times measured with either spike count algorithm (short-to-long: *F* = 0.063, *p* = 0.80; long-to-short: *F* = 0.20 *p* = 0.65).

**Table 2:** **Mean ± SE spike count recovery time (and range) as a function of cell type and level above threshold measured with the short-to-long and long-to-short analysis methods**.

**Cell type**	***n***	**Recovery time (ms) +10 dB**	***n***	**Recovery time (ms) +20 dB**
**SHORT-TO-LONG METHOD**				
Allpass	39	20.13 ± 2.93 (2–100)	32	17.50 ± 2.14 (0–54)
Bandpass	18	34.83 ± 5.82 (10–105)	13	23.54 ± 3.23 (6–42)
Shortpass	16	23.00 ± 3.81 (2–54)	14	18.79 ± 3.87 (2–54)
**LONG-TO-SHORT METHOD**				
Allpass	39	25.97 ± 4.05 (0–148)	32	20.91 ± 3.17 (0–76)
Bandpass	18	36.94 ± 5.64 (10–105)	13	26.15 ± 3.70 (6–44)
Shortpass	16	25.75 ± 4.49 (2–68)	14	20.21 ± 4.00 (2–50)

Recovery measured as the IPI where the spike count in response to P2 was ≥50% of the baseline spike count (re P1).

Tables 3 and 4 list the mean ± SE spike latency recovery times as a function of neural response class and level above threshold using the short-to-long and the long-to-short analysis methods. Overall, there was no main effect of response class or tone amplitude on FSL (Table 3) or LSL (Table 4) recovery times. Bandpass DTNs had significantly longer FSL recovery times with the long-to-short method (long-to-short FSL recovery: *F* = 3.90 *p* = 0.026). For the remainder of this paper, we present recovery times as the average of the short-to-long and long-to-short analysis algorithms for both spike counts and spike latencies unless we were unable to obtain an average, in which case the algorithm that provided a value was used.

**Table 3:** **Mean ± SE first-spike latency (FSL) recovery time (and range) as a function of cell type and level above threshold, measured with the short-to-long and long-to-short analysis methods**.

**Cell type**	***n***	**Recovery time (ms) +10 dB**	***n***	**Recovery time (ms) +20 dB**
**SHORT-TO-LONG METHOD**				
Allpass	36	20.64 ± 2.35 (4–58)	28	17.50 ± 2.05 (5–42)
Bandpass	17	23.65 ± 4.26 (2–66)	13	23.08 ± 5.36 (4–68)
Shortpass	16	21.56 ± 3.85 (4–50)	14	26.71 ± 6.40 (4–80)
**LONG-TO-SHORT METHOD**				
Allpass	29	23.52 ± 2.76 (8–60)	21	23.48 ± 2.52 (8–52)
Bandpass	14	41.14 ± 7.35 (10–110)	11	26.00 ± 5.67 (8–64)
Shortpass	14	34.50 ± 5.62 (10–70)	12	33.83 ± 6.76 (6–72)

Recovery time measured as the IPI where the FSL in response to P2 returned to within 1 SD of the baseline FSL (re P1).

**Table 4:** **Mean ± SE last-spike latency (LSL) recovery time (and range) as a function of cell type and level above threshold, measured with the short-to-long and long-to-short analysis methods**.

**Cell type**	***n***	**Recovery time (ms) +10 dB**	***n***	**Recovery time (ms) +20 dB**
**SHORT-TO-LONG METHOD**				
Allpass	20	13.65 ± 1.90 (4–45)	15	11.33 ± 2.09 (2–34)
Bandpass	13	18.08 ± 3.29 (2–45)	9	17.33 ± 3.46 (4–40)
Shortpass	9	15.33 ± 3.74 (4–42)	9	13.11 ± 1.74 (6–20)
**LONG-TO-SHORT METHOD**				
Allpass	15	18.40 ± 4.29 (6–58)	7	21.29 ± 5.45 (10–52)
Bandpass	6	42.67 ± 14.82 (12–110)	7	20.57 ± 4.31 (4–40)
Shortpass	9	16.44 ± 3.75 (6–42)	7	14.29 ± 4.52 (6–40)

Recovery time measured as the IPI where the LSL in response to P2 returned to within 1 SD of the baseline LSL (re P1).

**Figure 8:**
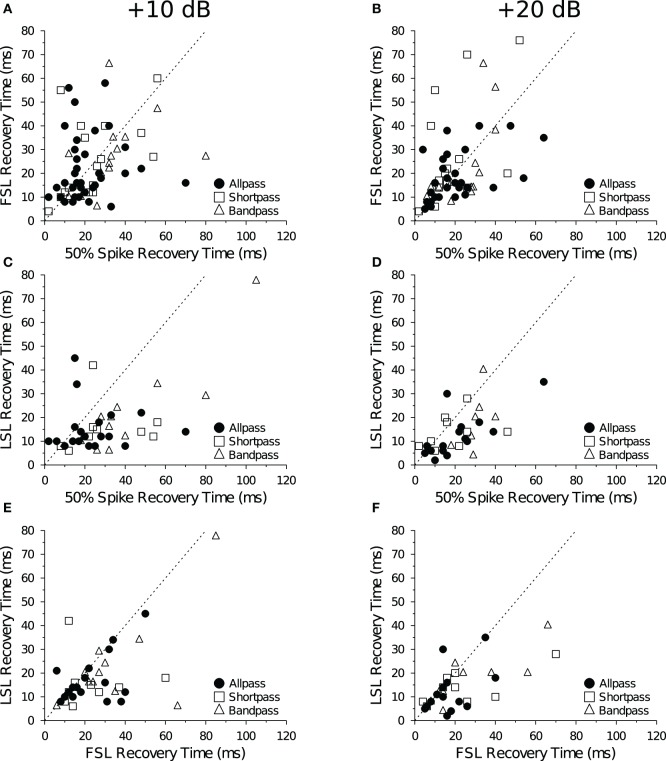
**Comparison of mean recovery times measured with spike counts and spike latencies.** Each panel shows the average recovery times of IC neurons at +10 dB (left) and +20 dB (right) re threshold. Recovery times are in agreement when points fall along the *dotted unity line y* = *x*. **(A,B)** There was a positive correlation between the mean 50% spike count and mean FSL recovery time in DTNs and non-DTNs at **(A)** +10 dB (*R*^2^ = 0.17, *p* < 0.001) and **(B)** +20 dB (*R*^2^ = 0.21, *p* < 0.001) re threshold. **(C,D)** There was a positive correlation between the mean spike count and mean LSL recovery time at **(C)** +10 dB (*R*^2^ = 0.28, *p* < 0.001) and **(D)** +20 dB (*R*^2^ = 0.35, *p* < 0.001) re threshold. **(E,F)** There was a positive correlation between FSL and LSL recovery times at **(E)** +10 dB (*R*^2^ = 0.32, *p* « 0.001) and **(F)** +20 dB (*R*^2^ = 0.41, *p* « 0.001) re threshold. The sample size (*n*) and number of points that fall above, below (but not on) the unity line are: **(A)** *n* = 69, 28 above, 39 below; **(B)** *n* = 55, 26 above, 24 below; **(C)** *n* = 42, 6 above, 35 below; **(D)** *n* = 34, 8 above, 25 below; **(E)** *n* = 42, 4 above, 25 below; and **(F)** *n* = 34, 4 above, 17 below.

Previous studies on the recovery cycle times of auditory neurons have used only spike counts as the dependent measure (Suga, 1964; Friend et al., 1966; Suga and Schlegel, 1973; Pollak et al., 1977b; Lu et al., 1997). In our dataset, we were able to measure recovery times with a 50% change in spike count for all 132 data files at +10 dB and +20 dB (re threshold). And for a majority of cells we were able to measure recovery time with a 1 SD change in FSL (*n* = 124 files) and LSL (*n* = 75 files). Recovery times measured with a spike count criterion were positively correlated with recovery times measured with a FSL (Figures 8A,B) and LSL (Figures 8C,D) criterion at both +10 dB and +20 dB (re threshold). There was also a positive correlation between recovery times measured with a FSL and LSL criterion at +10 dB (Figure 8E) and +20 dB above threshold (Figure 8F). Upon closer inspection, we found no obvious bias for recovery times measured with spike counts and FSLs because similar numbers of points fell above and below the unity lines at both levels above threshold (Figures 8A,B); however, recovery times measured with spike counts and FSLs tended to be longer than those measured with LSLs, as evidenced by a greater number of points falling below the unity lines in the scatterplots (Figures 8C–E).

In a further analysis we compared recovery times measured with spike counts, FSLs and LSLs but only in cells that provided values for all three measures (Table 5). Using this subset of data, the positive correlation between spike count and FSL recovery times remained and became stronger (*R*^2^ = 0.27, *p* < 0.001, *n* = 42). Measuring recovery times with a LSL criterion was a limiting factor for inclusion in this restricted subset of data, hence the correlations between spike count and LSL recovery times and between LSL and FSL recovery times were identical to those in Figures 8C–F. Using a repeated measures ANOVA, we found a significant main effect of the response parameter used to measure neural recovery (spike count, FSL, LSL: *F* = 15.02, *p* « 0.001). Recovery times determined with a 1 SD change in LSL were significantly shorter than recovery times measured with a 50% change in spike count or with a 1 SD change in FSL (Table 5). Moreover, there was now a significant main effect of stimulus amplitude, with recovery times decreasing at the higher stimulus level above threshold (+10 dB and +20 dB: *F* = 5.28, *p* = 0.024). There was no significant interaction between any main effects (analyses not shown).

**Table 5:** **Mean ± SE recovery times as a function of the measured recovery parameter and level above threshold**.

**Recovery parameter**	**Recovery time (ms) +10 dB**	**Recovery time (ms) +20 dB**
Spike count	33.38 ± 3.83	22.88 ± 2.30
FSL	25.14 ± 2.64	21.74 ± 2.82
LSL	17.99 ± 2.05	13.91 ± 1.61

Only cells with recovery times measured with all three parameters were included (n = 42: +10 dB; n = 34: +20 dB).

### 3.4. Recovery cycles and response properties

**Figure 9:**
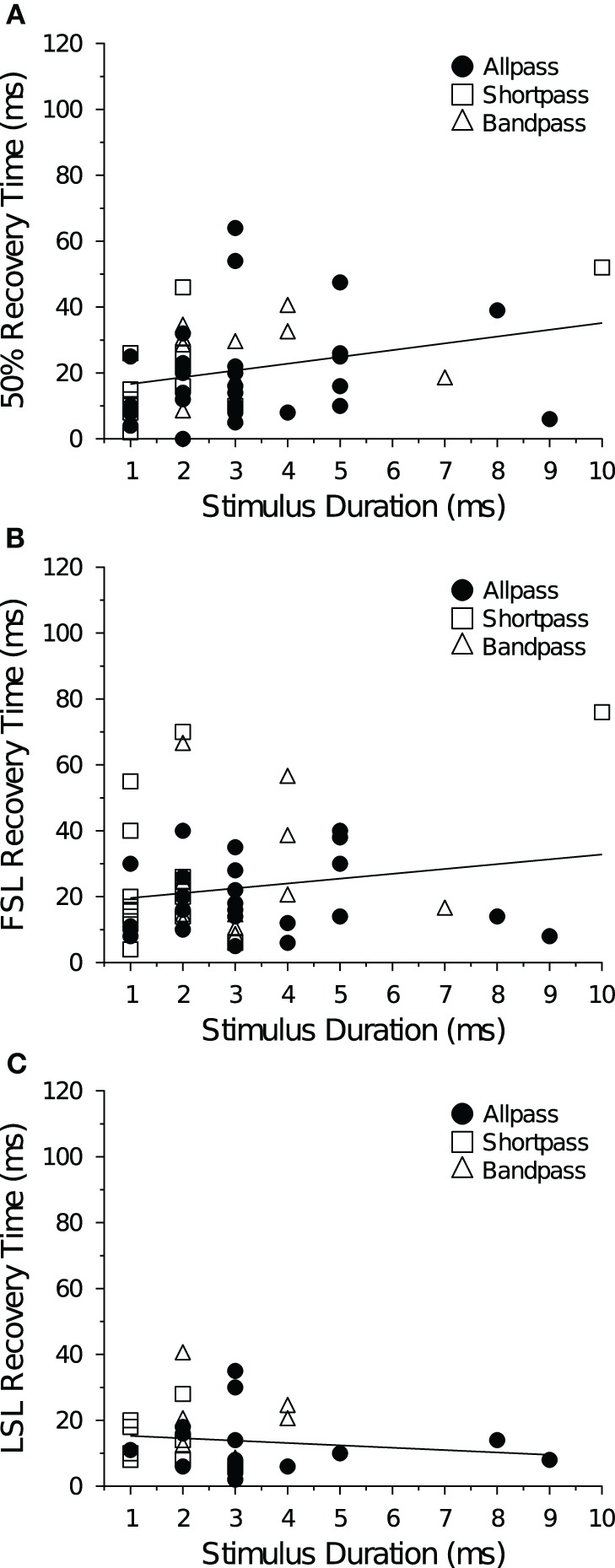
**Mean recovery time as a function of stimulus duration at +20 dB above threshold. (A)** Average 50% spike count recovery times as a function of paired tone stimulus duration. Spike count recovery times were positively correlated with stimulus duration (*R*^2^ = 0.081, *p* = 0.029). **(B)** Average FSL and **(C)** average LSL recovery times as a function of paired tone stimulus duration. Stimulus duration was not correlated with FSL (*R*^2^ = 0.030, *p* = 0.20) or LSL recovery times (*R*^2^ = 0.012, *p* = 0.54). Spike count, FSL and LSL recovery times were also not correlated with stimulus duration at +10 dB (re threshold). For DTNs stimulus duration was set to BD, whereas for allpass neurons stimulus duration was randomly chosen between 1 and 9 ms.

At +10 dB (re threshold), stimulus duration was not correlated with recovery cycle time, regardless of cell type (data not shown), but at +20 dB there was a positive correlation between stimulus duration and the spike count recovery time (Figure 9A). There was no correlation between stimulus duration and FSL recovery time (Figure 9B) or LSL recovery time (Figure 9C) at either level above threshold. When allpass neurons were removed from the analysis there was still no correlation between BD and the spike count recovery cycle time at +10 dB (*R*^2^ = 0.023, *p* = 0.39, *n* = 34), and the positive correlation at +20 dB became stronger (*R*^2^ = 0.16, *p* = 0.044, *n* = 26). Neural BEFs did not correlate with spike count recovery times at either +10 dB (*R*^2^ = 0.0019, *p* = 0.72) or +20 dB (*R*^2^ = 0.0095, *p* = 0.46) above threshold (data not shown). There was also no correlation between the spike count recovery time of a cell and its baseline spike count at both +10 dB (*R*^2^ = 0.019, *p* = 0.25) and +20 dB (*R*^2^ = 0.00024, *p* = 0.91) above threshold (data not shown).

**Figure 10:**
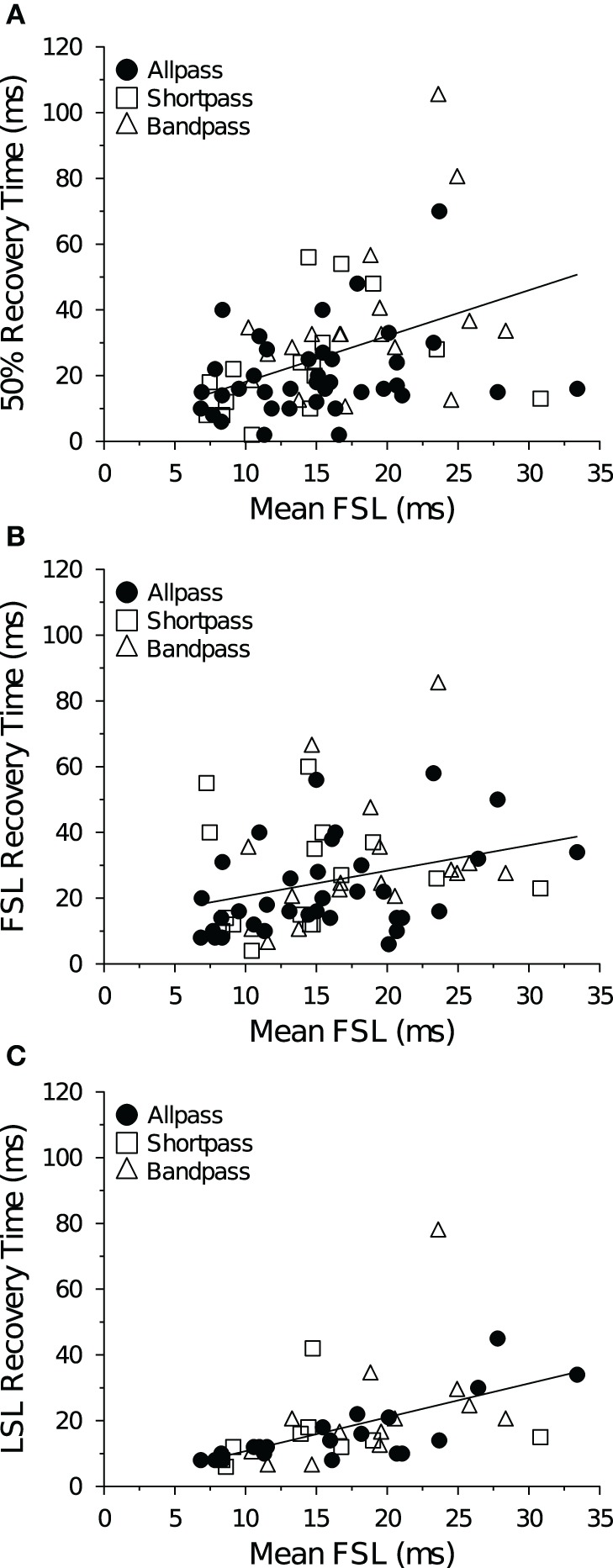
**Mean recovery time as a function of FSL at +10 dB above threshold. (A)** Average 50% spike count recovery times as a function of FSL. Neurons with longer FSLs had longer spike count recovery times (*R*^2^ = 0.16, *p* < 0.001, *n* = 73). **(B)** Average FSL and **(C)** average LSL recovery times as a function of FSL. Neurons with longer FSLs had longer FSL (*R*^2^ = 0.089, *p* = 0.013, *n* = 69) and LSL recovery times (*R*^2^ = 0.28, *p* < 0.001, *n* = 42). Spike count recovery times were also correlated with mean FSL at +20 dB (re threshold), but there was no correlation with FSL or LSL recovery times and mean FSL at +20 dB (re threhsold).

Neurons with short FSLs typically had short spike count recovery times, and this result held true at both +10 dB (Figure 10A) and +20 dB above threshold (data not shown). At +10 dB (re threshold), neurons with short FSLs also had short FSL (Figure 10B) and LSL recovery times (Figure 10C), but at +20 dB (re threshold) the correlations were no longer significant (data not shown).

## 4. Discussion

### 4.1. Recovery cycles and spike latencies

Spike counts and latencies are basic response properties commonly reported in electrophysiological papers. And while changes in both can be used to assess response features, such as the onset and duration of synaptic inhibition in DTNs (Faure et al., 2003) or the presence of neuromodulators in midbrain microcircuits (Hurley and Pollak, 2005), often studies report only a change in spike count even though numerous electrophysiological and computational papers have shown that spike latency is as (if not more) important at encoding stimulus specific and related information at all levels of the central auditory system (Middlebrooks et al., 1994; Klug et al., 2000; Brugge et al., 2001; Furukawa and Middlebrooks, 2002; Heil, 2004; Nelken et al., 2005). Fontaine and Peremans (2009) argue that a spike-timing code (as opposed to a spike rate or count code) is more appropriate for processing the short duration echolocation signals emitted by bats. Neural FSLs may also be used as an alternative to spike counts/rates when rapid responses are required (Grothe and Klump, 2000; VanRullen et al., 2005), and are critical to mechanisms underlying sound localization (Joris et al., 1998).

In this study, we compared recovery cycle times measured with spike counts and spike latencies. We do not know which measures are more pertinent for assessing neural response recovery and/or its relevance to perception because both spike count and timing codes can be used independently to represent stimulus features (VanRullen et al., 2005). The relative importance, if any, will depend on which parameter(s) is(are) most important for encoding and transmitting stimulus specific information in the central nervous system. Because our analyses employed dissimilar criteria for assessing response recovery (50% spike count *versus* 1 SD latency change), the sets of results may not be directly comparable. Despite this caveat, recovery times measured with spike counts were reasonably well correlated with those measured with spike latencies (Figure 8). Nevertheless, some neurons with small spike count recovery times had large spike latency recovery times (and vice versa), suggesting that some factors governing spike count recovery differ from those governing spike latency recovery. When we restricted our analysis and examined only those cells that provided recovery times with spike count, FSL and LSL, the correlations between the recovery times remained (or strengthened). Recovery times measured with LSL were significantly shorter than those measured with spike counts and FSL in the same cells (Table 5). Altogether, the results suggest that spike latencies can be employed as an alternative measure of response recovery. We encourage researchers to continue developing additional analyses that exploit changes in spike latency.

Inhibition is thought to play a role in determining FSL and the duration of recovery times of central auditory neurons. Bicucculine (GABA_A_ receptor antagonist) application shortens the recovery times of most IC neurons (Lu et al., 1997; Zhou and Jen, 2003) and also shorten FSLs (Park and Pollak, 1993; Lu et al., 1997). The effect of inhibition on FSL has been disputed. It has been suggested that a reduction in FSL can be attributed to offset responding neurons changing to onset responding neurons when inhibition is removed or its effects are blocked because this causes FSL to shorten by the duration of the stimulus (Fuzessery et al., 2003). Intracellular recordings from the IC of the big brown bat reveal the presence of an onset-evoked hyperpolarization in the majority of units studied (Covey et al., 1996; Voytenko and Galazyuk, 2008), suggesting that onset-evoked inhibition plays a role in governing first-spike timing. In this study we found that neurons with shorter FSLs had shorter recovery times, a relationship that also exists for sound localizing neurons in the IC of the awake rabbit (Fitzpatrick et al., 1995). Together, these findings support the hypothesis that factors influencing the duration of recovery cycles and the timing of FSLs are related.

### 4.2. Effect of stimulus amplitude and BEF on FSL and recovery times

As in previous studies, we found that FSLs of DTNs and non-DTNs in the IC of the bat decreased with increasing electrode depth (Park and Pollak, 1993; Fuzessery et al., 2003) and increasing BEFs (Haplea et al., 1994; Fuzessery et al., 2003). Neural FSLs also decreased with increasing stimulus amplitude (Table 3; Heil, 2004; Tan et al., 2008). We found no correlation between neuronal BEFs and spike count recovery times at either +10 dB or +20 dB (re threshold). This result differs from a previous study that found a strong negative correlation between these variables in the IC of the bat (Zhou and Jen, 2003). Gross electrode recordings from the bat's auditory brainstem revealed that stimulation with higher acoustic frequencies resulted in faster response recovery (Grinnell, 1963). Given that FSL is also negatively correlated with BEF (Figure 3; Haplea et al., 1994; Fuzessery et al., 2003), if a negative correlation exists between BEF and spike count recovery time then we might expect a similar correlation between FSL and spike count recovery time. In the present study, we were unable to detect a correlation between BEF and the spike count recovery time at either level above threshold. Therefore, the positive relationship that we observed between spike count recovery time and FSL cannot be explained by a covariance with BEF.

Some studies have used pairs of unequal amplitude tones to determine the recovery cycle characteristics of IC neurons. For example, Friend et al. (1966) found that recovery times shortened as the intensity of stimulus P1 was decreased relative to P2. This result is consistent with the hypothesis that inhibition evoked by P1 was easier to overcome by increased excitation evoked by the relatively higher intensity P2 stimulus. Moreover, the effect of stimulus amplitude on spike count recovery times co-varies with stimulus duration (Wang et al., 2008, 2010). Using gross electrode recordings in the bat brainstem in response to equal amplitude tones, Grinnell (1963) reported more rapid neural recovery for stimuli of reduced intensity—a finding that is opposite to our results. In the present study, recovery times in the population of DTNs and non-DTNs remained stable over a 10 dB change in SPL in response to pairs of equal amplitude tones (Tables 2–4); however, when we restricted our analysis to look only at those cells with recovery times measured with spike count and latency criteria, recovery times shortened from +10 to +20 dB (re threshold) regardless of the recovery parameter examined (Table 5). This suggests that neural excitation may have increased relative to inhibition at higher levels above threshold. Additional studies employing a wider range of stimulus amplitudes are needed to determine if the recovery times of DTNs are tolerant over the same dynamic range (40–50 dB) as the amplitude tolerance of duration tuning (Zhou and Jen, 2001; Fremouw et al., 2005).

### 4.3. Spatial mapping of best duration

We found no correlation between neuronal BD and the depth of the recording electrode in the population of DTNs tested (Figure 2). This result agrees with a number of previous studies that found no spatial organization of DTNs in the ICc of the bat (Pinheiro et al., 1991; Ehrlich et al., 1997; Faure et al., 2003; Luo et al., 2008). In contrast, other studies have reported a significant positive correlation between BD and BEF (Jen and Wu, 2006; Wu and Jen, 2006, 2008b), suggesting the possibility that the ICc of the bat contains a spatial map of duration tuning. In the present study, we found a significant negative correlation between BD and BEF (Figure 2)—a relationship in the opposite direction of three previous reports. Given the inconsistency of the result within and across laboratories, we conclude there is no strong evidence to support the hypothesis that a spatial map of duration tuning exists in the IC of the bat.

### 4.4. Biological significance of recovery times

Recovery cycle times of IC neurons in the cat (Yin, 1994; Litovsky and Yin, 1998a,b), rabbit (Fitzpatrick et al., 1995, 1999) and barn owl (Keller and Takahashi, 1996) have been suggested as a potential neural mechanism underlying the precedence effect for humans listening in reverberant environments (for review, see Litovsky et al., 1999). The precedence effect is a binaural psychoacoustical phenomenon that describes the sound localization performance of participants listening to pairs of sounds separated by an interval (e.g., pulse and reflected echo). When two sounds have a brief IPI (<1 ms; Wallach et al., 1949), participants detect a single sound located in a position midway between the sources (i.e., summing localization). When two sounds are presented with a longer delay (1–5 ms IPI for single clicks; ≤35–70 ms IPI for other complex sounds; Wallach et al., 1949), participants localize the source in the direction of the first sound. Because the first arriving wavefront takes precedence in sound localization, the precedence effect is also known as the law of the first wavefront. Recovery cycle times measured from auditory neurons in the ICc of the bat (Tables 2–4) broadly correlate with the range of IPIs over which the precedence effect occurs in humans. A behavioural correlate of the precedence effect has been studied in cats (3–16 ms; Cranford and Oberholtzer, 1976), rats (0.25–16 ms; Kelly, 1974), and crickets (4–75 ms; Wyttenbach and Hoy, 1993). While it seems reasonable to assume that echolocating bats also experience a precedence effect during acoustic orientation and prey detection, psychoacoustical experiments on two species of gleaning bats suggest they may not (Schuchmann et al., 2006).

Our results demonstrate that recovery cycle times of midbrain auditory neurons in the bat, which have been explored mainly in the context of echolocation (for review, see Jen et al., 2012), are quite similar to those measured from the IC of other vertebrates (Yin, 1994; Fitzpatrick et al., 1995; Keller and Takahashi, 1996). This suggests that factors governing the recovery cycle times of central auditory neurons are similar in echolocating and non-echolocating species. It also reinforces the utility of bats as an animal model for understanding general principles of mammalian hearing and auditory physiology despite the fact that bats echolocate and are “hearing specialists.” The function(s) of DTNs to hearing and echolocation is(are) still unknown; however, the range of neural BDs and the temporal bandwidth of duration tuning are generally matched to the range of vocalization durations in echolocating bats (Sayegh et al., 2011). If DTNs play a direct role in echolocation, then one might predict they should exhibit short recovery times so that the same neuron could respond to both loud outgoing vocalizations and later returning echoes. Alternatively, owing to the importance of leading and persistent inhibition in creating temporally selective neural responses, one could also predict that the inhibition evoked by each pulse in a paired tone stimulus would temporally interact and sum, resulting in DTNs exhibiting longer recovery cycle times than non-DTNs. Our results support the latter prediction: bandpass DTNs had significantly longer recovery times than shortpass DTNs and allpass neurons. In contrast, a recent study examining auditory neurons in the IC of the echolocating bat *Pipistrellus abramus* found no significant difference in the recovery times of DTNs and allpass neurons (Wang et al., 2010).

If DTNs function in echo ranging, then the recovery cycle time would play an important role in determining the minimum target distance over which a neuron could respond. The recovery time can be viewed as equivalent to the two-way travel time of the bat's outgoing sound and returning echo. Assuming a recovery cycle time for a DTN of 36 ms, this would be equivalent to a one-way travel time of 18 ms (speed of sound in air = 344 mm/ms) and would correspond to a target distance of 6.19 m (344 mm/ms*18 ms). Neurons with shorter (longer) recovery cycles would have shorter (longer) minimum target detection distances because these cells could respond more rapidly (slowly) to successive sound presentations such as pulse-echo pairs. For example, a neuron with a recovery time of 2 ms could fire action potentials in response to both the pulse and echo at a minimum distance of 34.4 cm (344 mm/ms*1 ms), whereas a neuron with a recovery time of 100 ms could encode a minimum target distance of 17.2 m (344 mm/ms*50 ms).

In theory, the recovery time of a DTN, in combination with BD, could determine perceptual integration time and cause a cell to be tuned to detect small pulse-echo delays and short target distances. For example, assume a bandpass DTN with a BD of 5 ms is stimulated with pulse and echo biosonar sounds lasting 2 ms each. Although the cell may not spike in response to either the pulse (P1) or the echo (P2) when presented alone, simply because the durations of both signals are shorter than the cell's BD, the neuron might respond to the stimulus pair if presented at a short gap of 1 ms representing a target at 17.2 cm (344 mm/ms*0.5 ms). In this case, the DTN may respond to the pulse-echo pair as a single, unified (i.e., fused) stimulus because the combined 2 ms pulse + 1 ms gap + 2 ms echo duration is close to the neuronal BD (Sayegh et al., 2011). By extending this line of reasoning we would predict that neurons with short BDs would be tuned to short target distances, and neurons with longer BDs would be tuned to longer target distances. This is because cells with long BDs may be able to tolerate a wider range of silence between pulse-echo pairs at sub-optimal durations. In support of this novel hypothesis, which is distinct but not mutually exclusive from the idea that DTNs are tuned to the duration of single relevant sounds, we found a weak but positive correlation between stimulus duration and spike count recovery time at +20 dB above threshold (Figure 9B), but there was no correlation between stimulus duration and spike latency recovery times. Grinnell (1963) observed an increase in recovery time as the duration of the P1 (but not P2) stimulus increased. Jen et al. (2012) reported that spike count recovery times of DTNs increased with the duration of the pulse-echo stimulus; however, this effect does not hold for equal amplitude stimulus pairs and occurs only in some neurons at specific pulse-echo amplitude differences (Wang et al., 2008). In contrast, Pollak et al. (1977a) found recovery times of IC auditory neurons increased at short (<2 ms) stimulus durations. These conflicting reports point to the need for future studies to test the hypothesis that DTNs can respond to pairs of tones presented at sub-optimal durations (i.e., not at BD) and separated by relatively short gaps of silence.

In a small subset of cells we observed a brief facilitation in the spike count ratio of recovery function at short IPIs (Figures 6C,D). These neurons could be delay-tuned combination-sensitive cells that respond best at short echo delays (O'Neill and Suga, 1982; Portfors and Wenstrup, 1999). The range of recovery times that we observed for both DTNs and non-DTNs in the ICc of *E. fuscus* nicely corresponds to the range of pulse-to-pulse intervals (repetition rates) used by foraging bats during the search (50–100 ms), approach (10–50 ms) and terminal feeding buzz (4–7 ms) phases of hunting (Griffin et al., 1960). The subset of DTNs with facilitatory responses at short IPIs may be especially well-suited for echolocation at short target distances.

### 4.5. Recovery cycles and neural inhibition

Faure et al. (2003) used paired tone stimulation to measure the onset and duration of the leading and persistent inhibition that creates DTNs in the IC of the bat. In that study, P1 was set to the neuron's BD and P2 was set to a longer, non-excitatory duration. In the present study, we used pairs of excitatory tones to measure and compare the recovery cycle times of DTNs with non-DTNs, and found that bandpass DTNs had significantly longer recovery times than non-DTNs using spike count recovery and one measure of spike latency recovery. These results also highlight the importance of neural inhibition to duration tuning. Previous studies have suggested that the strength and time course of GABAergic inhibition can account for some variation in the recovery cycles of DTNs. Blocking GABAergic inhibition with bicucculine shortened recovery times in a majority of IC cells (Lu et al., 1997; Zhou and Jen, 2003). Presenting stimuli that mimicked pulse-echo pairs at short IPIs also sharpened duration-selectivity (Wu and Jen, 2006; Jen and Wu, 2008) and frequency-selectivity (Wu and Jen, 2008a,b); in the latter two examples, the sharpening of the response evoked by the “echo” was thought to have arisen from the recruitment and persistence of inhibition evoked by the “pulse.”

During paired tone stimulation, if the inhibition evoked by stimulus P1 encroaches upon the excitation evoked by stimulus P2 then recovery times of cells with persistent inhibition are expected to be longer than in cells with less or no persistent inhibition. We might also expect FSLs (re signal onset) to increase because spikes evoked by P2 could be delayed by persistent inhibition evoked by P1. Meanwhile, neuronal LSLs (re P2) may largely be unaffected. In general, our results support the hypothesis that recovery cycle times are determined, at least in part, by the inhibition evoked by tone P1 that persists and influences the spiking responses evoked by tone P2. We also found that FSLs (re P2) were more likely to deviate by >1 SD from baseline than LSLs (re P2), a finding that is consistent with the effects of persistent inhibition.

Recovery times measured in IC neurons are on the order of tens of milliseconds and therefore cannot be due to a neuron's absolute and/or relative refractory periods caused by post-spiking increases in potassium permeability and inactivation of sodium channels because these effects typically last only a few milliseconds (Hodgkin, 1951; Hodgkin and Huxley, 1952). Stimulus repetition rate can also affect the measurement of recovery times. In the ICc of the bat, increasing stimulus repetition rate increases the observed FSL and minimum threshold (Jen and Chen, 1998), increases directional selectivity (Zhou and Jen, 2004), and alters duration selectivity (Jen and Feng, 1999; Jen and Wu, 2005; Zhou and Jen, 2006). In theory, increasing the stimulus repetition rate increases the recovery cycle time of a neuron because persistent inhibition evoked by one stimulus trial influences responses evoked on the subsequent trial. In our study, stimulus repetition rate cannot explain differences in the recovery cycle times between DTNs and non-DTNs because all of the data were collected at the same rate (3 Hz). We believe that differences in the recovery times between DTNs and non-DTNs were caused, in part, by inhibition lasting longer than the duration of the P1 tone that evoked it. This persistent inhibition would sum with the leading inhibition evoked by tone P2, resulting in DTNs exhibiting longer recovery times than a random selection of other types of IC neurons not tuned to stimulus duration. Other factors can also affect neural recovery times, including the length of the axons that provide synaptic input (axonal delay; Smith et al., 1993), the time course of temporal facilitation and depression of excitatory and inhibitory synapses (e.g., neurotransmitter depletion; Zucker and Regehr, 2002), receptor desensitization (Raman and Trussell, 1992; Raman et al., 1994), presynaptic modulation of inhibition via intracellular calcium accumulation (Lu and Trussell, 2000) and/or GABA_B_ receptors (Ma et al., 2002), and intrinsic cellular properties such as subthreshold sound-evoked oscillations (Hechavarra et al., 2011). Additional studies are needed to shed light on the mechanisms that shape and govern the recovery cycle times of mammalian central auditory neurons.

## 5. Summary

1. Spike counts are traditionally used to measure the recovery cycle times of neurons. Herein we demonstrate that spike latencies may also be used to measure response recovery. In general, recovery times measured with spike counts were positively correlated with recovery times measured with spike latencies, although recovery times measured with a 1 SD change in LSL (re baseline latency) were significantly shorter than recovery times measured with a 50% change in spike count (re baseline count) or with a 1 SD change in FSL (re baseline latency).
2. Previous studies have shown that neural inhibition is necessary for creating DTNs in the IC of the bat. Because DTNs are known to have inhibition that persists for as long or longer than the duration of the stimulus evoking the inhibition, we predicted that DTNs would have longer recovery times than non-DTNs. Recovery times of bandpass DTNs obtained with spike counts and one measure of FSL recovery were longer than recovery times of shortpass DTNs and non-DTNs.
3. Increasing the amplitude of the paired tone stimulus from +10 to +20 dB (re threshold) did not shorten recovery times in the population of cells tested, indicating that recovery kinetics of IC neurons in the bat are tolerant to a +10 dB change in stimulus amplitude. When we restricted our analysis to the subset of neurons that provided recovery time values measured with spike counts, FSLs, and LSLs, we found that increasing stimulus amplitude shortened recovery times in the same cells. Additional studies employing a wider range of stimulus levels are needed to fully characterize the effect of stimulus amplitude on the recovery times of IC neurons.
4. Neurons with short FSLs had shorter recovery times than cells with longer FSLs. Inhibition is an important determinant of the recovery cycle time and FSL of DTNs and non-DTNs. Our results demonstrate that the neural mechanisms controlling FSL and recovery cycle kinetics may be related.

### Conflict of interest statement

The authors declare that the research was conducted in the absence of any commercial or financial relationships that could be construed as a potential conflict of interest.

## References

[B1] Aubie B. Becker S. Faure P. A. (2009). Computational models of millisecond level duration tuning in neural circuits. J. Neurosci. 29, 9255–9270 10.1523/JNEUROSCI.1085-09.2009 19625516 PMC6665553

[B2] Aubie B. Sayegh R. Faure P. A. (2012). Duration tuning across vertebrates. J. Neurosci. 32, 6373–6390 10.1523/JNEUROSCI.5624-11.2012 22553042 PMC6622122

[B3] Brand A. Urban A. Grothe B. (2000). Duration tuning in the mouse auditory midbrain. J. Neurophysiol. 84, 1790–1799 11024071 10.1152/jn.2000.84.4.1790

[B4] Brugge J. F. Reale R. A. Jenison R. L. Schnupp J. (2001). Auditory cortical spatial receptive fields. Audiol. Neurootol. 6, 173–177 11694722 10.1159/000046827

[B5] Carr C. E. Konishi M. (1990). A circuit for detection of interaural time differences in the brain stem of the barn owl. J. Neurosci. 10, 3227–3246 2213141 10.1523/JNEUROSCI.10-10-03227.1990 PMC6570189

[B6] Casseday J. H. Ehrlich D. Covey E. (1994). Neural tuning for sound duration: role of inhibitory mechanisms in the inferior colliculus. Science 264, 847–850 10.1126/science.8171341 8171341

[B7] Casseday J. H. Ehrlich D. Covey E. (2000). Neural measurement of sound duration: control by excitatory-inhibitory interactions in the inferior colliculus. J. Neurophysiol. 84, 1475–1487 10980020 10.1152/jn.2000.84.3.1475

[B8] Chen G.-D. (1998). Effects of stimulus duration on responses of neurons in the chinchilla inferior colliculus. Hear. Res. 112, 142–150 10.1016/S0378-5955(98)00103-8 9714582

[B9] Covey E. Kauer J. A. Casseday J. H. (1996). Whole-cell patch-clamp recording reveals subthreshold sound-evoked postsynaptic currents in the inferior colliculus of awake bats. J. Neurosci. 16, 3009–3018 8622130 10.1523/JNEUROSCI.16-09-03009.1996 PMC6579070

[B10] Cranford J. L. Oberholtzer M. (1976). Role of neocortex in binaural hearing in the cat. II. The ‘precedence effect’ in sound localization. Brain Res. 111, 225–239 10.1016/0006-8993(76)90768-X 949598

[B11] Denes P. (1955). Effect of duration on the perception of voicing. J. Acoust. Soc. Am. 27, 761–764

[B12] Duysens J. Schaafsma S. J. Orban G. A. (1996). Cortical off response tuning for stimulus duration. Vision Res. 36, 3243–3251 10.1016/0042-6989(96)00040-5 8944284

[B13] Ehrlich D. Casseday J. H. Covey E. (1997). Neural tuning to sound duration in the inferior colliculus of the big brown bat, *Eptesicus fuscus*. J. Neurophysiol. 77, 2360–2372 9163363 10.1152/jn.1997.77.5.2360

[B14] Faingold C. L. Boersma Anderson C. A. Caspary D. M. (1991). Involvement of GABA in acoustically-evoked inhibition in inferior colliculus neurons. Hear. Res. 52, 201–216 2061208 10.1016/0378-5955(91)90200-s

[B15] Faure P. A. Fremouw T. Casseday J. H. Covey E. (2003). Temporal masking reveals properties of sound-evoked inhibition in duration-tuned neurons of the inferior colliculus. J. Neurosci. 23, 3052–3065 12684492 10.1523/JNEUROSCI.23-07-03052.2003 PMC6742117

[B16] Faure P. A. Re D. E. Clare E. L. (2009). Wound healing in the flight membranes of big brown bats. J. Mammal. 90, 1148–1156

[B17] Fitzpatrick D. C. Kuwada S. Batra R. Trahiotis C. (1995). Neural responses to simple simulated echoes in the auditory brain stem of the unanesthetized rabbit. J. Neurophysiol. 74, 2469–2486 8747207 10.1152/jn.1995.74.6.2469

[B18] Fitzpatrick D. C. Kuwada S. Kim D. O. Parham K. Batra R. (1999). Responses of neurons to click-pairs as simulated echoes: auditory nerve to auditory cortex. J. Acoust. Soc. Am. 106, 3460–3472 10.1121/1.428199 10615686

[B19] Fontaine B. Peremans H. (2009). Bat echolocation processing using first-spike latency coding. Neural Netw. 22, 1372–1382 10.1016/j.neunet.2009.05.002 19481904

[B20] Frederiksen E. (1977). Condenser microphones used as sound sources. Brüel and Kjær Tech. Rev. 3, 3–23

[B21] Fremouw T. Faure P. A. Casseday J. H. Covey E. (2005). Duration selectivity of neurons in the inferior colliculus of the big brown bat: tolerance to changes in sound level. J. Neurophysiol. 94, 1869–1878 10.1152/jn.00253.2005 15888527

[B22] Friend J. H. Suga N. Suthers R. A. (1966). Neural responses in the inferior colliculus of echolocating bats to artificial orientation sounds and echoes. J. Cell. Physiol. 67, 319–332 10.1002/jcp.1040670212 5924098

[B23] Furukawa S. Middlebrooks J. C. (2002). Cortical representation of auditory space: information-bearing features of spike patterns. J. Neurophysiol. 87, 1749–1762 10.1152/jn.00491.2001 11929896

[B24] Fuzessery Z. M. Hall J. C. (1999). Sound duration selectivity in the pallid bat inferior colliculus. Hear. Res. 137, 137–154 10.1016/S0378-5955(99)00133-1 10545641

[B25] Fuzessery Z. M. Wenstrup J. J. Hall J. C. Leroy S. (2003). Inhibition has little effect on response latencies in the inferior colliculus. JARO 4, 60–73 10.1007/s10162-002-2054-6 12183767 PMC3202449

[B26] Gooler D. M. Feng A. S. (1992). Temporal coding in the frog auditory midbrain: the influence of duration and rise-fall time on the processing of complex amplitude-modulated stimuli. J. Neurophysiol. 67, 1–22 1552312 10.1152/jn.1992.67.1.1

[B27] Griffin D. R. Webster F. A. Michael C. R. (1960). The echolocation of flying insects by bats. Anim. Behav. 8, 141–154

[B28] Grinnell A. D. (1963). The neurophysiology of audition in bats: temporal parameters. J. Physiol. 167, 67–96 13950554 10.1113/jphysiol.1963.sp007133 PMC1359485

[B29] Grothe B. Klump G. M. (2000). Temporal processing in sensory systems. Curr. Opin. Neurobiol. 10, 467–473 10981615 10.1016/s0959-4388(00)00115-x

[B30] Haplea S. Covey E. Casseday J. H. (1994). Frequency tuning and response latencies at three levels in the brainstem of the echolocating bat, *Eptesicus fuscus*. J. Comp. Physiol. A 174, 671–683 8014918 10.1007/BF00192716

[B31] He J. Hashikawa T. Ojima H. Kinouchi Y. (1997). Temporal integration and duration tuning in the dorsal zone of cat auditory cortex. J. Neurosci. 17, 2615–2625 9065521 10.1523/JNEUROSCI.17-07-02615.1997 PMC6573496

[B32] Hechavarra J. C. Cobo A. T. Fernández Y. Macas S. Kössl M. Mora E. C. (2011). Sound-evoked oscillation and paradoxical latency shift in the inferior colliculus of the big fruit-eating bat, *Artibeus jamaicensis*. J. Comp. Physiol. A 197, 1159–1172 10.1007/s00359-011-0678-x 21912875

[B33] Heil P. (2004). First-spike latency of auditory neurons revisited. Curr. Opin. Neurobiol. 14, 461–467 10.1016/j.conb.2004.07.002 15321067

[B34] Hodgkin A. L. (1951). The ionic basis of electrical activity in nerve and muscle. Biol. Rev. 26, 339–409

[B35] Hodgkin A. L. Huxley A. F. (1952). A quantitative description of membrane current and its application to conduction and excitation in nerve. J. Physiol. 117, 500–544 12991237 10.1113/jphysiol.1952.sp004764 PMC1392413

[B36] Hurley L. M. Pollak G. D. (2005). Serotonin shifts first-spike latencies of inferior colliculus neurons. J. Neurosci. 25, 7876–7886 10.1523/JNEUROSCI.1178-05.2005 16120790 PMC6725259

[B37] Jen P. H.-S. Chen Q.-C. (1998). The effect of pulse repetition rate, pulse intensity, and bicuculline on the minimum threshold and latency of bat inferior collicular neurons. J. Comp. Physiol. A 182, 455–465 10.1007/s003590050193 9530836

[B38] Jen P. H.-S. Feng R. B. (1999). Bicuculline application affects discharge pattern and pulse-duration tuning characteristics of bat inferior collicular neurons. J. Comp. Physiol. A 184, 185–194 10.1007/s003590050317 10192952

[B39] Jen P. H.-S. Schlegel P. A. (1982). Auditory physiological properties of the neurones in the inferior colliculus of the big brown bat, *Eptesicus fuscus*. J. Comp. Physiol. A 147, 351–363

[B40] Jen P. H.-S. Wu C. H. (2005). The role of GABAergic inhibition in shaping the response size and duration selectivity of bat inferior collicular neurons to sound pulses in rapid sequences. Hear. Res. 202, 222–234 10.1016/j.heares.2004.11.008 15811714

[B41] Jen P. H.-S. Wu C. H. (2006). Duration selectivity organization in the inferior colliculus of the big brown bat, *Eptesicus fuscus*. Brain Res. 1108, 76–87 10.1016/j.brainres.2006.06.017 16828465

[B42] Jen P. H.-S. Wu C. H. (2008). Echo duration selectivity of the bat varies with pulse-echo amplitude difference. Neuroreport 19, 373–377 10.1097/WNR.0b013e3282f52c61 18303584

[B43] Jen P. H.-S. Wu C. H. Wang X. (2012). Dynamic temporal signal processing in the inferior colliculus of echolocating bats. Front. Neural Circuits 6:27 10.3389/fncir.2012.00027 22586374 PMC3347223

[B44] Jen P. H.-S. Zhou X. M. (1999). Temporally patterned pulse trains affect duration tuning characteristics of bat inferior collicular neurons. J. Comp. Physiol. A 185, 471–478 10.1007/s003590050408 10573869

[B45] Joris P. X. Smith P. H. Yin T. C. T. (1998). Coincidence detection in the auditory system: 50 years after Jeffress. Neuron 21, 1235–1238 10.1016/S0896-6273(00)80643-1 9883717

[B46] Keller C. H. Takahashi T. T. (1996). Responses to simulated echoes by neurons in the barn owl's auditory space map. J. Comp. Physiol. A 178, 499–512 8847663 10.1007/BF00190180

[B47] Kelly J. B. (1974). Localization of paired sound sources in the rat: small time differences. J. Acoust. Soc. Am. 55, 1277–1284 10.1121/1.1914697 4846722

[B48] Klug A. Bauer E. E. Pollak G. D. (1999). Multiple components of ipsilaterally evoked inhibition in the inferior colliculus. J. Neurophysiol. 82, 593–610 10444659 10.1152/jn.1999.82.2.593

[B49] Klug A. Khan A. Burger R. M. Bauer E. E. Hurley L. M. Yang L. Grothe B. Halvorsen M. B. Park T. J. (2000). Latency as a function of intensity in auditory neurons: influences of central processing. Hear. Res. 148, 107–123 10.1016/S0378-5955(00)00146-5 10978829

[B50] Knudsen E. I. Konishi M. (1979). Mechanisms of sound localization in the barn owl (*Tyto alba*). J. Comp. Physiol. A 133, 13–21

[B51] Kuwada S. Batra R. Yin T. C. T. Oliver D. L. Haberly L. B. Stanford T. R. (1997). Intracellular recordings in response to monaural and binaural stimulation of neurons in the inferior colliculus of the cat. J. Neurosci. 17, 7565–7581 9295401 10.1523/JNEUROSCI.17-19-07565.1997 PMC6573453

[B52] Leary C. J. Edwards C. J. Rose G. J. (2008). Midbrain auditory neurons integrate excitation and inhibition to generate duration selectivity: an *in vivo* whole-cell patch study in anurans. J. Neurosci. 28, 5481–5493 10.1523/JNEUROSCI.5041-07.2008 18495882 PMC2582375

[B53] Litovsky R. Y. Colburn H. S. Yost W. A. Guzman S. J. (1999). The precedence effect. J. Acoust. Soc. Am. 106, 1633–1654 10.1121/1.427914 10530009

[B54] Litovsky R. Y. Delgutte B. (2002). Neural correlates of the precedence effect in the inferior colliculus: effect of localization cues. J. Neurophysiol. 87, 976–994 11826062 10.1152/jn.00568.2001

[B55] Litovsky R. Y. Yin T. C. T. (1998a). Physiological studies of the precedence effect in the inferior colliculus of the cat. I. Correlates of psychophysics. J. Neurophysiol. 80, 1285–1301 9744939 10.1152/jn.1998.80.3.1285

[B56] Litovsky R. Y. Yin T. C. T. (1998b). Physiological studies of the precedence effect in the inferior colliculus of the cat. II. Neural mechanisms. J. Neurophysiol. 80, 1302–1316 9744940 10.1152/jn.1998.80.3.1302

[B57] Lu T. Trussell L. O. (2000). Inhibitory transmission mediated by asynchronous transmitter release. Neuron 26, 683–694 10.1016/S0896-6273(00)81204-0 10896163

[B58] Lu Y. Jen P. H.-S. Zheng Q.-Y. (1997). GABAergic disinhibition changes the recovery cycle of bat inferior collicular neurons. J. Comp. Physiol. A 181, 331–341 10.1007/s003590050119 9342856 PMC2862906

[B59] Luo F. Metzner W. Wu F. J. Zhang S. Y. Chen Q. C. (2008). Duration-sensitive neurons in the inferior colliculus of horseshoe bats: adaptations for using CF-FM echolocation pulses. J. Neurophysiol. 99, 284–296 10.1152/jn.00935.2007 18003879

[B60] Ma C. L. Kelly J. B. Wu S. H. (2002). Presynaptic modulation of GABAergic inhibition by GABA_B_ receptors in the rat's inferior colliculus. Neuroscience 114, 207–215 10.1016/S0306-4522(02)00130-6 12207966

[B61] Middlebrooks J. C. Clock A. E. Xu L. Green D. M. (1994). A panoramic code for sound location by cortical neurons. Science 264, 842–844 10.1126/science.8171339 8171339

[B62] Mora E. C. Kössl M. (2004). Ambiguities in sound duration selectivity by neurons in the inferior colliculus of the bat *Molossus molossus* from Cuba. J. Neurophysiol. 91, 2215–2226 10.1152/jn.01127.2003 14711975

[B63] Nelken I. Chechik G. Mrsic-Flogel T. D. King A. J. Schnupp J. W. H. (2005). Encoding stimulus information by spike numbers and mean response time in primary auditory cortex. J. Comput. Neurosci. 19, 199–221 10.1007/s10827-005-1739-3 16133819

[B64] O'Neill W. E. Suga N. (1982). Encoding of target range and its representation in the auditory cortex of the mustached bat. J. Neurosci. 2, 17–31 7054393 10.1523/JNEUROSCI.02-01-00017.1982 PMC6564294

[B65] Park T. J. Pollak G. D. (1993). GABA shapes a topographic organization of response latency in the mustache bat's inferior colliculus. J. Neurosci. 13, 5172–5187 8254367 10.1523/JNEUROSCI.13-12-05172.1993 PMC6576431

[B66] Pedemonte M. Torterolo P. Velluti R. A. (1997). *In vivo* intracellular characteristics of inferior colliculus neurons in guinea pigs. Brain Res. 759, 24–31 10.1016/S0006-8993(97)00123-6 9219859

[B67] Pérez-González D. Malmierca M. S. Moore J. M. Hernández O. Covey E. (2006). Duration selective neurons in the inferior colliculus of the rat: topographic distribution and relation of duration sensitivity to other response properties. J. Neurophysiol. 95, 823–836 10.1152/jn.00741.2005 16192332

[B68] Pinheiro A. D. Wu M. Jen P. H.-S. (1991). Encoding repetition rate and duration in the inferior colliculus of the big brown bat, *Eptesicus fuscus*. J. Comp. Physiol. A 169, 69–85 1941720 10.1007/BF00198174

[B69] Pinheiro J. C. Bates D. M. (2000). Mixed-Effects Models in S and S-PLUS. (New York, NY: Springer-Verlag).

[B70] Pollack G. S. Hoy R. R. (1979). Temporal pattern as a cue for species-specific calling song recognition in crickets. Science 204, 429–432 10.1126/science.204.4391.429 17758018

[B71] Pollak G. D. Bodenhamer R. Marsh D. S. Souther A. (1977a). Recovery cycles of single neurons in the inferior colliculus of unanesthetized bats obtained with frequency-modulated and constant-frequency sounds. J. Comp. Physiol. A 120, 215–250

[B72] Pollak G. D. Marsh D. S. Bodenhamer R. Souther A. (1977b). Characteristics of phasic on neurons in inferior colliculus of unanesthetized bats with observations relating to mechanisms for echo ranging. J. Neurophysiol. 40, 926–942 886375 10.1152/jn.1977.40.4.926

[B73] Pollak G. D. Park T. J. (1993). The effects of GABAergic inhibition on monaural response properties of neurons in the mustache bat's inferior colliculus. Hear. Res. 65, 99–117 8384613 10.1016/0378-5955(93)90205-f

[B74] Portfors C. V. Wenstrup J. J. (1999). Delay-tuned neurons in the inferior colliculus of the mustached bat: implications for analyses of target distance. J. Neurophysiol. 82, 1326–1338 10482752 10.1152/jn.1999.82.3.1326

[B75] Potter H. D. (1965). Patterns of acoustically evoked discharges of neurons in the mesencephalon of the bullfrog. J. Neurophysiol. 28, 1155–1184 5883734 10.1152/jn.1965.28.6.1155

[B76] Raman I. M. Trussell L. O. (1992). The kinetics of the response to glutamate and kainate in neurons of the avian cochlear nucleus. Neuron 9, 173–186 10.1016/0896-6273(92)90232-3 1352983

[B77] Raman I. M. Zhang S. Trussell L. O. (1994). Pathway-specific variants of AMPA receptors and their contribution to neuronal signaling. J. Neurosci. 14, 4998–5010 7913958 10.1523/JNEUROSCI.14-08-04998.1994 PMC6577204

[B78] Sayegh R. Aubie B. Faure P. A. (2011). Duration tuning in the auditory midbrain of echolocating and non-echolocating vertebrates. J. Comp. Physiol. A 197, 571–583 10.1007/s00359-011-0627-8 21305304

[B79] Schuchmann M. Hübner M. Wiegrebe L. (2006). The absence of spatial echo suppression in the echolocating bats *Megaderma lyra* and *Phyllostomus discolor*. J. Exp. Biol. 209, 152–157 10.1242/jeb.01975 16354786

[B80] Simmons J. A. (1971). Echolocation in bats: signal processing of echoes for target range. Science 171, 925–928 10.1126/science.171.3974.925 5541661

[B81] Simmons J. A. (1979). Perception of echo phase information in bat sonar. Science 204, 1336–1338 10.1126/science.451543 451543

[B82] Smith P. H. Joris P. X. Yin T. C. T. (1993). Projections of physiologically characterized spherical bushy cell axons from the cochlear nucleus of the cat: evidence for delay lines to the medial superior olive. J. Comp. Neurol. 331, 245–260 10.1002/cne.903310208 8509501

[B83] Stapells D. R. Picton T. W. Smith A. D. (1982). Normal hearing thresholds for clicks. J. Acoust. Soc. Am. 72, 74–79 10.1121/1.388026 7108045

[B84] Suga N. (1964). Recovery cycles and responses to frequency modulated tone pulses in auditory neurones of echo-locating bats. J. Physiol. 175, 50–80 14241158 10.1113/jphysiol.1964.sp007503 PMC1357085

[B85] Suga N. O'Neill W. E. (1979). Neural axis representing target range in the auditory cortex of the mustache bat. Science 206, 351–353 10.1126/science.482944 482944

[B86] Suga N. Schlegel P. (1973). Coding and processing in the auditory systems of FM-signal-producing bats. J. Acoust. Soc. Am. 54, 174–190 10.1121/1.1913561 4731643

[B87] Tan M. L. Borst J. G. G. (2007). Comparison of responses of neurons in the mouse inferior colliculus to current injections, tones of different durations, and sinusoidal amplitude-modulated tones. J. Neurophysiol. 98, 454–466 10.1152/jn.00174.2007 17507505

[B88] Tan X. Wang X. Yang W. Xiao Z. (2008). First spike latency and spike count as functions of tone amplitude and frequency in the inferior colliculus of mice. Hear. Res. 235, 90–104 10.1016/j.heares.2007.10.002 18037595

[B89] Tang J. Fu Z.-Y. Jen P. H.-S. Chen Q.-C. (2011). Recovery cycles of single-on and double-on neurons in the inferior colliculus of the leaf-nosed bat, *Hipposideros armiger*. Brain Res. 1385, 114–126 10.1016/j.brainres.2011.02.031 21338589

[B90] Torterolo P. Pedemonte M. Velluti R. A. (1995). Intracellular *in vivo* recording of inferior colliculus auditory neurons from awake guinea-pigs. Arch. Ital. Biol. 134, 57–64 8919192

[B91] VanRullen R. Guyonneau R. Thorpe S. J. (2005). Spike times make sense. Trends Neurosci. 28, 1–4 10.1016/j.tins.2004.10.010 15626490

[B92] Voytenko S. V. Galazyuk A. V. (2008). Timing of sound-evoked potentials and spike responses in the inferior colliculus of awake bats. Neuroscience 155, 923–936 10.1016/j.neuroscience.2008.06.031 18621102 PMC2577224

[B93] Wallach H. Newman E. B. Rosenzweig M. R. (1949). The precedence effect in sound localization. Am. J. Psychol. 62, 315–336 18134356

[B94] Wang J. van Wijhe R. Chen Z. Yin S. (2006). Is duration tuning a transient process in the inferior colliculus of guinea pigs? Brain Res. 1114, 63–74 10.1016/j.brainres.2006.07.046 16919248

[B95] Wang X. Luo F. Jen P. H.-S. Chen Q.-C. (2010). Recovery cycle of neurons in the inferior colliculus of the FM bat determined with varied pulse-echo duration and amplitude. Chinese J. Physiol. 53, 119–129 21793319 10.4077/cjp.2010.amk030

[B96] Wang X. Luo F. Wu F.-J. Chen Q.-C. Jen P. H. S. (2008). The recovery cycle of bat duration-selective collicular neurons varies with hunting phase. Neuroreport 19, 861–865 10.1097/WNR.0b013e3282ffb574 18463502

[B97] Wu C. H. Jen P. H.-S. (2006). GABA-mediated echo duration selectivity of inferior collicular neurons of *Eptesicus fuscus*, determined with single pulses and pulse-echo pairs. J. Comp. Physiol. A 192, 985–1002 10.1007/s00359-006-0133-6 16738883

[B98] Wu C. H. Jen P. H.-S. (2008a). Auditory frequency selectivity is better for expected than for unexpected sound duration. Neuroreport 19, 127–131 10.1097/WNR.0b013e3282f3b11c 18281906

[B99] Wu C. H. Jen P. H.-S. (2008b). Echo frequency selectivity of duration-tuned inferior collicular neurons of the big brown bat, *Eptesicus fuscus*, determined with pulse-echo pairs. Neuroscience 156, 1028–1038 10.1016/j.neuroscience.2008.08.039 18804149

[B100] Wyttenbach R. A. Hoy R. R. (1993). Demonstration of the precedence effect in an insect. J. Acoust. Soc. Am. 94, 777–784 10.1121/1.408207 8370884

[B101] Xia Y.-F. Qi Z.-H. Shen J.-X. (2000). Neural representation of sound duration in the inferior colliculus of the mouse. Acta Otolaryngol. 120, 638–643 11039876 10.1080/000164800750000478

[B102] Yin T. C. T. (1994). Physiological correlates of the precedence effect and summing localization in the inferior colliculus of the cat. J. Neurosci. 14, 5170–5186 8083729 10.1523/JNEUROSCI.14-09-05170.1994 PMC6577094

[B103] Zhou X. Jen P. H.-S. (2001). The effect of sound intensity on duration-tuning characteristics of bat inferior collicular neurons. J. Comp. Physiol. A 187, 63–73 11318379 10.1007/s003590000179

[B104] Zhou X. Jen P. H.-S. (2003). The effect of bicuculline application on azimuth-dependent recovery cycle of inferior collicular neurons of the big brown bat, *Eptesicus fuscus*. Brain Res. 973, 131–141 12729962 10.1016/s0006-8993(03)02575-7

[B105] Zhou X. Jen P. H.-S. (2004). Azimuth-dependent recovery cycle affects directional selectivity of bat inferior collicular neurons determined with sound pulses within a pulse train. Brain Res. 1019, 281–288 10.1016/j.brainres.2004.06.004 15306265

[B106] Zhou X. Jen P. H.-S. (2006). Duration selectivity of bat inferior collicular neurons improves with increasing pulse repetition rate. Chinese J. Physiol. 49, 46–55 16900705

[B107] Zucker R. S. Regehr W. G. (2002). Short-term synaptic plasticity. Annu. Rev. Physiol. 64, 355–405 10.1146/annurev.physiol.64.092501.114547 11826273

